# Leveraging browse and grazing forage estimates to optimize index-based livestock insurance

**DOI:** 10.1038/s41598-024-62893-4

**Published:** 2024-06-27

**Authors:** Njoki Kahiu, J. Anchang, V. Alulu, F. P. Fava, N. Jensen, N. P. Hanan

**Affiliations:** 1 New Mexico State University, Las Cruces, USA; 2 International Livestock Research Institute (ILRI), Nairobi, Kenya; 3 Department of Environmental Science and Policy (ESP), Università degli Studi di Milano, Milan, Italy; 4 University of Edinburgh, Edinburgh, Scotland

**Keywords:** Aggregate leaf area index (LAI_A_), Herbaceous leaf area index (LAI_H_), Index Based Livestock Insurance (IBLI), Livestock mortality, Normalized Difference Vegetation Index (NDVI), Woody leaf area index (LAI_W_), Climate sciences, Environmental sciences, Environmental social sciences, Natural hazards

## Abstract

African pastoralists suffer recurrent droughts that cause high livestock mortality and vulnerability to climate change. The index-based livestock insurance (IBLI) program offers protection against drought impacts. However, the current IBLI design relying on the normalized difference vegetation index (NDVI) may pose limitation because it does not consider the mixed composition of rangelands (including herbaceous and woody plants) and the diverse feeding habits of grazers and browsers. To enhance IBLI, we assessed the efficacy of utilizing distinct browse and grazing forage estimates from woody LAI (LAI_W_) and herbaceous LAI (LAI_H_), respectively, derived from aggregate leaf area index (LAI_A_), as an alternative to NDVI for refined IBLI design. Using historical livestock mortality data from northern Kenya as reference ground dataset, our analysis compared two competing models for (1) aggregate forage estimates including sub-models for NDVI, LAI (LAI_A_); and (2) partitioned biomass model (LAI_P_) comprising LAI_H_ and LAI_W_. By integrating forage estimates with ancillary environmental variables, we found that LAI_P_, with separate forage estimates, outperformed the aggregate models. For total livestock mortality, LAI_P_ yielded the lowest RMSE (5.9 TLUs) and higher R^2^ (0.83), surpassing NDVI and LAI_A_ models RMSE (9.3 TLUs) and R^2^ (0.6). A similar pattern was observed for species-specific livestock mortality. The influence of environmental variables across the models varied, depending on level of mortality aggregation or separation. Overall, forage availability was consistently the most influential variable, with species-specific models showing the different forage preferences in various animal types. These results suggest that deriving distinct browse and grazing forage estimates from LAI_P_ has the potential to reduce basis risk by enhancing IBLI index accuracy.

## Introduction

Pastoralists living in arid and semiarid lands (ASALS) of Africa primarily depend on livestock for their livelihoods^1^. Livestock play a significant role in generating income and employment, supplying nutrients, supporting cultural practices, providing resilience against economic and climate shocks and supporting crop production in agropastoral systems^1^. However, the persistent vulnerability of pastoral livelihoods to recurring and intense drought events, the leading cause of livestock mortality^2,3^, poses a formidable challenge, adversely impacting a substantial pastoral population in the region.

The implications of livestock mortality are multifaceted, encompassing the depletion of household assets, disruption of livelihoods, compromised nutritional security, loss of valuable genetic resources, and the loss of substantial investments, encompassing both financial resources and labor^4^. This vulnerability is further exacerbated by escalating climate variability, mounting population pressure, and rangeland degradation^5^, which collectively diminish the effectiveness of traditional herding strategies, such as migration, and strain the informal coping mechanisms of pastoral societies, thereby exacerbating poverty^6^. The recent emergence of index-based livestock insurance (IBLI) has begun to provide much-needed respite, shielding pastoralists against the adverse impacts of drought^7–9^. Index-based insurance products are primarily used as financial tools for mitigating risks associated with agriculture and livestock, particularly those stemming from natural disasters^10^. Unlike traditional insurance products, index insurance, often referred to as parametric insurance, operates on a unique principle where indemnity is not determined by actual losses but relies on the empirical relationship between a chosen proxy index and the expected loss associated with the covered risk^11,12^. The selection of these proxy indices is crucial and depends on the specific risk being addressed. Some of the common proxy indices encompass weather information and Earth Observation (EO) derived vegetation yield information. The index of choice should be strongly correlated with the risk being modeled, to provide a reliable basis for triggering insurance payouts when predetermined thresholds or conditions are met^12^. Presently, IBLI operates in parts of Kenya and Ethiopia as part of commercial and government subsidized program^13^, with expansion initiatives underway across other parts of Africa^14–16^. In the current setup IBLI is defined to cover extreme drought conditions whereby payouts are made when forage availability falls below predefined thresholds, thus policyholders receive compensation they can use to mitigate the impact of drought related adversities on their livelihoods.

IBLI operates on the principle of indemnification, utilizing a predetermined external variable known as the "*forage index*" derived from EO data, strongly correlated with drought and forage scarcity, rather than depending on individual loss assessments, which can be both time-consuming and potentially biased^17^. This approach renders IBLI efficient, credible, accessible and immune to potential manipulations by various stakeholders. IBLI is characterized by its cost-effectiveness and independence from data on actual livestock mortality, which can be financially and logistically difficult to acquire in many African regions^6,18^. Consequently, the index-based approach provides an accessible insurance option for poor and vulnerable pastoralists who may lack formal documentation or face challenges in proving their losses, thus providing them with a reliable safety net during drought which would otherwise be impossible in conventional insurance markets^19,20^. The fundamental mechanism underlying IBLI involves automatic payouts triggered when the forage index falls below a predefined threshold within a geographical unit designed to capture shared risk and referred to as the unit area of insurance (UAI). A UAI is defined as an area with homogeneous biophysical or agroclimatic characteristics, similar pastoral experiences, socioeconomic factors and risk profile^21,22^. Neighboring pastoral communities are grouped into the same UAI cluster based on these criteria.

An additional strength of the IBLI approach lies in the ability to provide payments early in the season, allowing pastoralists to proactively safeguard their livestock before the onset of significant mortality, reducing reliance on less favorable coping strategies^18^. Beyond its immediate benefits, IBLI addresses the root causes of climate-related vulnerability among pastoral communities and holds the potential to catalyze economic growth, enhance household food security, and bolster resilience among marginalized pastoralists^23^. Moreover, IBLI has been instrumental in catalyzing insurance markets across numerous African countries over the past decade. It has made previously unaffordable or nonexistent insurance options accessible. This has the capacity to attract private sector investment and augment both public and private capital flows, contribute to the improvement of financial institutions and infrastructures, fostering a more robust financial ecosystem^18,24^.

While IBLI has emerged as an effective tool in mitigating the impacts of drought on pastoral and crop production systems^6,25^, it only provides partial protection^26^ and faces a significant challenge in the form of potential basis risk. The basis risk represents the discrepancy between the index-triggered indemnity payments and actual observed losses^6,12^. Product design in IBLI encompasses several critical elements that directly influence insurance payouts, including the degree of correlation between the chosen index and average available forage, formulation of payout models, and the established trigger thresholds. For instance, in the current IBLI formulation, given that different livestock species exhibit distinct feeding preferences, the chosen index should aptly reflect the dietary habits of the covered animal, ensuring relevance and accuracy in assessing risks and potential losses. These disparities can lead to a mismatch between the index used for insurance and the actual risk being covered, predominantly drought.

The existing IBLI contract relies on a satellite measurement of vegetation greenness, the Normalized Difference Vegetation Index (NDVI), which serves as a proxy for forage availability by measuring vegetation vigor. NDVI is aggregated over UAIs, encompassing diverse rangeland types to gauge forage status relative to long-term average seasonal profiles^17^. However, livestock in pastoral systems largely feed on herbaceous biomass, hence limiting the applicability of aggregate NDVI for estimating forage availability in rangelands in mixed tree grass systems. Moreover, intended IBLI expansion across regions in Africa, including a diversity of agro-silvopastoral systems, poses a challenge in the current contract design due to heterogeneity in land cover, with areas of denser woody cover, mixed crop and rangeland vegetation. This is exacerbated by ongoing invasion of non-palatable species^27^ and woody encroachment in pastoral lands^28^, some of which remain evergreen year-round, further complicating forage estimation, particularly during drought.

This research explored the use of novel satellite-derived data products for the estimation of the forage index in the context of IBLI product design. The newly considered inputs were based on MODIS leaf area index (LAI_A_; defined as the area of green leaves per unit ground area, an indicator of foliage quantity) partitioned into herbaceous (LAI_H_) and woody (LAI_W_) forage components. Thus, the objective of this research was to evaluate the feasibility and effectiveness of utilizing distinct woody (LAI_W_; a proxy for browsing resources) and herbaceous (LAI_H_; a proxy for grazing resources) forage estimates as an alternative to the commonly used NDVI for the enhancement of IBLI product design. To test these approaches, we utilized historical livestock mortality data from northern Kenya as a reference dataset for index accuracy assessment and employed a random forest regression framework to examine two competing forage type models: (1) the aggregate biomass model which represents the mixed woody (shrubs and trees) and herbaceous (grasses and forbs) foliage including sub-models for (a) NDVI model, (b) LAI model (LAI_A_), and (2) partitioned LAI model (LAI_P_), comprising separate woody (LAI_W_) and herbaceous (LAI_H_) forage estimates. The distribution of livestock and their ability to survive drought in African rangelands are influenced by a complex interplay of forage availability and other ecological, climatic, socio-economic, and management factors. Environmental factors, including ecological and climatic elements, are relatively straightforward to represent using EO derived metrics. In contrast, socio-economic and management factors may not always be readily accessible for analysis^2,6^. Hence, within the framework of this analysis, we integrated the forage availability proxies with other pertinent environmental variables, including water availability, temperature, and the extent of human landscapes, to elucidate the factors influencing livestock mortality in Kenyan pastoral rangelands.

## Materials and methods

### Study area

**Figure 1:**
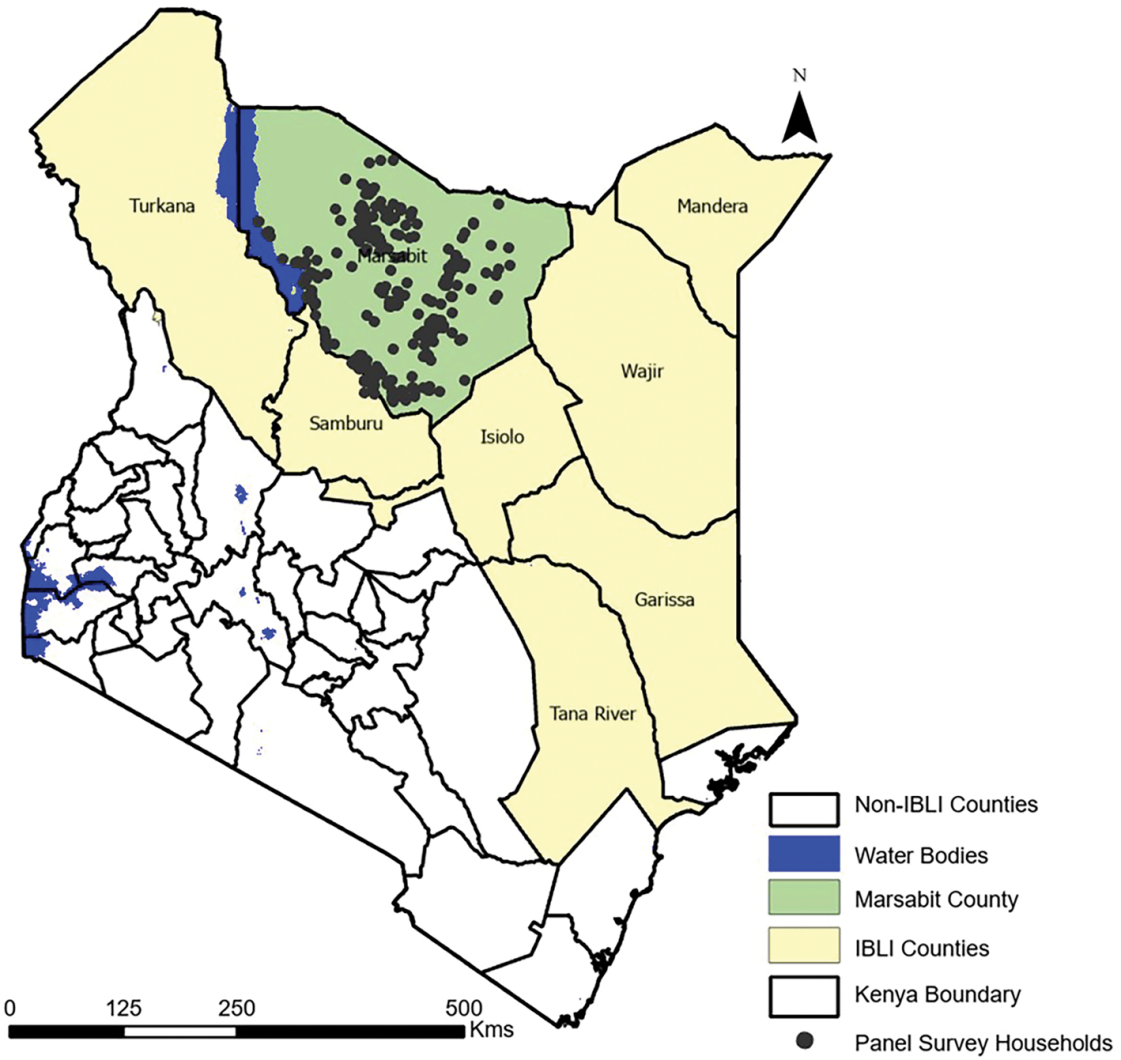
Map of Kenya, showing the study area of Marsabit county and the Counties participating in the index-based livestock insurance (IBLI) program. Approximate location of household surveys is shown for Marsabit County, conducted in different seasons on livestock status, losses from drought mortality and other events. Figure generated in ArcGIS Pro 3.2.2.

The study location was in Marsabit County, northern Kenya, where a time series of livestock mortality data were collected between 2009 and 2021 (Fig. 1). The county is one of Kenya’s arid and semi-arid regions where IBLI has been implemented, with mean annual precipitation (MAP) 200–300 mm across most of the County, with some small localized mountainous areas receiving up to 800 mm annually^29,30^. Rainfall follows the typical bimodal precipitation pattern of the Horn of Africa (HoA), with the long rainy season (LRS) occurring from March to June, followed by a three-month dry period and the short rain season (SRS) from October to December. Daytime temperatures vary in the range 22–35 °C between the cold and hot months.

Marsabit County is among the Kenya's largest counties in terms of land area, covering ~ 12% of the country’s total landmass, yet it is sparsely populated with a total population of 459,785 individuals, translating to approximately 6 persons/km^2^^31^. The population is ethnically diverse, encompassing groups pastoral and non-pastoral communities^30,31^.

Pastoralism is the main source of livelihood among the various communities in the county, who practice both sedentary and nomadic pastoralism. Marsabit County, like many other arid and semi-arid areas in the region, contends with recurrent droughts occurring every 2–3 years, some of which extend beyond a single season, resulting in various drought related challenges. Over the years, the county has grappled with livestock mortality due to drought, conflicts arising from competition for scarce foraging resources among pastoralists, and an upsurge in disease outbreaks when foraging resources become critically low, leading to mass livestock migrations and congregations in the few available grazing reserves^30,32^. Additionally, cattle raids are a common occurrence, with neighboring communities invading during wetter seasons mainly to recover livestock lost in the aftermath of preceding droughts^30^.

### Data and pre-processing steps

Marsabit observes a bimodal rainfall pattern which corresponds with two distinct forage production periods, which are commonly used to define insurance coverage risk periods for IBLI^2,6^. The severity of drought-related livestock mortality can vary between these seasons due to differences in their duration and behavior. To account for this variability, our analysis categorized seasons based on static monthly definitions. We combined the wet season with the subsequent dry period to address the lag in livestock mortality caused by forage shortages during the wet season^33^. Thus, we defined the LRS (March–June) and the following dry season (July–September) as the first seven-month mortality season, termed the long rains long dry (LRLD; March to September). The short rains (October–December) and two-month dry period that follows (January–February) were then referred to as the short rains and short dry (SRSD; October–February). These seasonal timelines served as the basis for defining drought-related mortality statistics and derivation of vegetation indices^29^.

#### Livestock mortality

The livestock mortality data comprised panel surveys of pastoralist households in Marsabit county (Fig. 1). The data was collected as part of the International Livestock Research Institute (ILRI) IBLI initiative for the epoch 2008–2021. It comprised a randomized control trial of pastoral communities with the overarching goal of representing the diverse pastoral populations in northern Kenya. The data collected included livestock status data such as household stock sizes, details on livestock losses (including assumed causes), livestock inflows and outflows, income, expenditure, and population demographics^34^.

Longitudinal data were collected in October/November of 2009, 2010, 2011, 2012, 2013, 2015, and 2021 for this analysis across a total of 1027 households. While there were slight variations in household participation across the seven rounds of data collection, the majority of households consistently took part in these repeat surveys. During the annual surveys, respondents were asked recall questions on specific livestock-related events including losses, intakes, offtakes, slaughters, and births, all within the context of the 12 months preceding each survey, with reference to a specific month of the year^34^. The primary data were collected on camels, cattle, sheep, and goats. During the surveys, sheep and goats were typically grouped together under the term *"shoats"*. To address concerns related to data retrieval from memory and potential inaccuracies in mortality reports, efforts were made to identify and eliminate any apparent data duplications and inconsistencies.

The analysis was conducted at the household level, leveraging the availability of GPS location data for each household. This enabled us to extract and incorporate EO derived vegetation and environmental variables at household level in our analysis. Pastoralists migrate across vast areas of land in search of foraging resources^35^. The range of pastoral migration is influenced by ecological, climatic, and socio-economic factors, and it varies across different regions of Africa^36^. Here we assumed that most pastoralists in northern Kenya have access to forage within a 20 km radius of their communal grazing lands, although this range may expand during extreme droughts^37,38^. This assumption holds true, especially in eastern Africa where land fragmentation due to land use changes has diminished available grazing lands. Thus, for each household we created a buffer of 20 km, then used it to extract the EO derived vegetation indices and other environmental variables used in the analysis.

To calculate mortality rates associated with droughts in our analysis, we included mortality losses stemming from both drought and disease (Table 1). We argued that, droughts in pastoral systems limit forage and water resources, leading to reduced nutrition quality and quantity for livestock, increasing their susceptibility to diseases and increasing mortality rates^36,39^. Additionally, disease outbreaks are more prevalent during dry seasons when livestock congregate around water sources and limited foraging resources, increasing disease transmission between herds, further elevating mortality rates^32^.

**Table 1:** Reported causes of livestock loss in the Marsabit household surveys and their reclassification for use in the current analysis.

	Loss reason	Loss reclassification
1	Accident/poisoned	Other
2	Disease	Disease
3	Lost	Other
4	Old age	Other
5	Predation	Predation
6	Premature birth	Other
7	Raiding/rustling/conflict	Conflict
8	Rain	Drought
9	Starvation/drought	Drought
10	Other (specify as consumed plastic bags, premature birth, bloat, ceremony, birth complication, snake bite	Other

To match seasonal estimates of livestock mortality and vegetation indices used as proxies for forage availability we did a seasonal aggregation of mortality. To establish uniformity in quantifying livestock across our models, we employed the conversion of individual mortality figures into Tropical Livestock Units (TLU). TLU serves as a standardized metric for expressing the size or relative value of various livestock species in tropical regions. It is anchored on a ruminant with a liveweight of 250 kg, typically representing an adult cow^40^. For consistency, we adopted the TLU conversion rates established by ILRI for the implementation of IBLI in northern Kenya, as outlined in Table 2.

**Table 2:** Tropical livestock units conversion factors used for implementation of index-based livestock insurance in northern Kenya.

Livestock species	TLU conversion rate
Cattle	1
Sheep	0.1
Goats	0.1
Camels	1.3

#### Indicators of forage availability and water resources

The availability of water, forage resources and their nutritional quality are key determinants of livestock distribution. Access to sufficient and nutritious forage is critical for livestock survival, especially during drought periods. Overgrazing, land degradation, extreme weather patterns and climate variability can reduce forage availability, thus significantly impacting livestock distribution and drought survival in African rangelands. Areas with more reliable rainfall tend to have higher available forage thus can support larger livestock populations. During critical periods relocation to grazing reserves is critical for livestock survival. Thus, in this analysis we used EO derived vegetation indices including NDVI and leaf area index (LAI) as proxies for forage availability across seasons. Furthermore, the presence of reliable water sources plays a crucial role in livestock survival during droughts and affects pastoral grazing patterns. Areas with access to water are the best foraging zones as most livestock have a high dependency on water. Hence, we used EO derived seasonal water estimates as an indicator of water availability.

##### Aggregate leaf area index (LAI_A_)

In this analysis we used EO derived LAI as a proxy for landscape-scale aggregate forage resources, which constitutes mixed grazing (herbaceous) and browse (from woody vegetation) resources. LAI provides valuable information about the amount of green vegetation cover and foliage area. LAI is a dimensionless parameter, defined as the one-sided area of green leaves (m^2^) per unit ground area (m^2^) in broadleaf canopies and half total needle surface area per unit ground area in conifers. LAI values range from 0 (no vegetation) to values exceeding 6 in dense vegetation^41^. Here, we used Moderate Resolution Imaging Spectroradiometer (MODIS) Collection 6.1 (MC6.1) LAI from NASA's Terra and Aqua satellites to estimate total landscape-scale LAI comprising mixed vegetation components for woody and herbaceous vegetation (“aggregate LAI”, denoted as *LAI*_*A*_). This distinction serves to differentiate it from its derivatives that separate the aggregate LAI into woody (LAI_W_) and herbaceous (LAI_H_) LAI constituents, which form what we term the partitioned LAI (LAI_P_), as presented in the section that follows. This dataset offers comprehensive coverage at 8-day intervals with a spatial resolution of 500 m^42^ from 2002 to 2021.

Following pre-processing based on MODIS quality flags^43^ we used the approach detailed in Kahiu and Hanan^44^ to filter out cloud-contaminated pixels, fill data gaps and reduce noise, including a robust spline smoothing algorithm implemented in Python^45^. Despite the MODIS satellite nearing the end of its operational life^46^, we opted for this data source due to its extensive historical archive, which aligns with the livestock mortality data spanning from 2008 to 2021. This choice allowed us to establish a lengthy reference period for the computation of the forage index.

**Figure 2:**
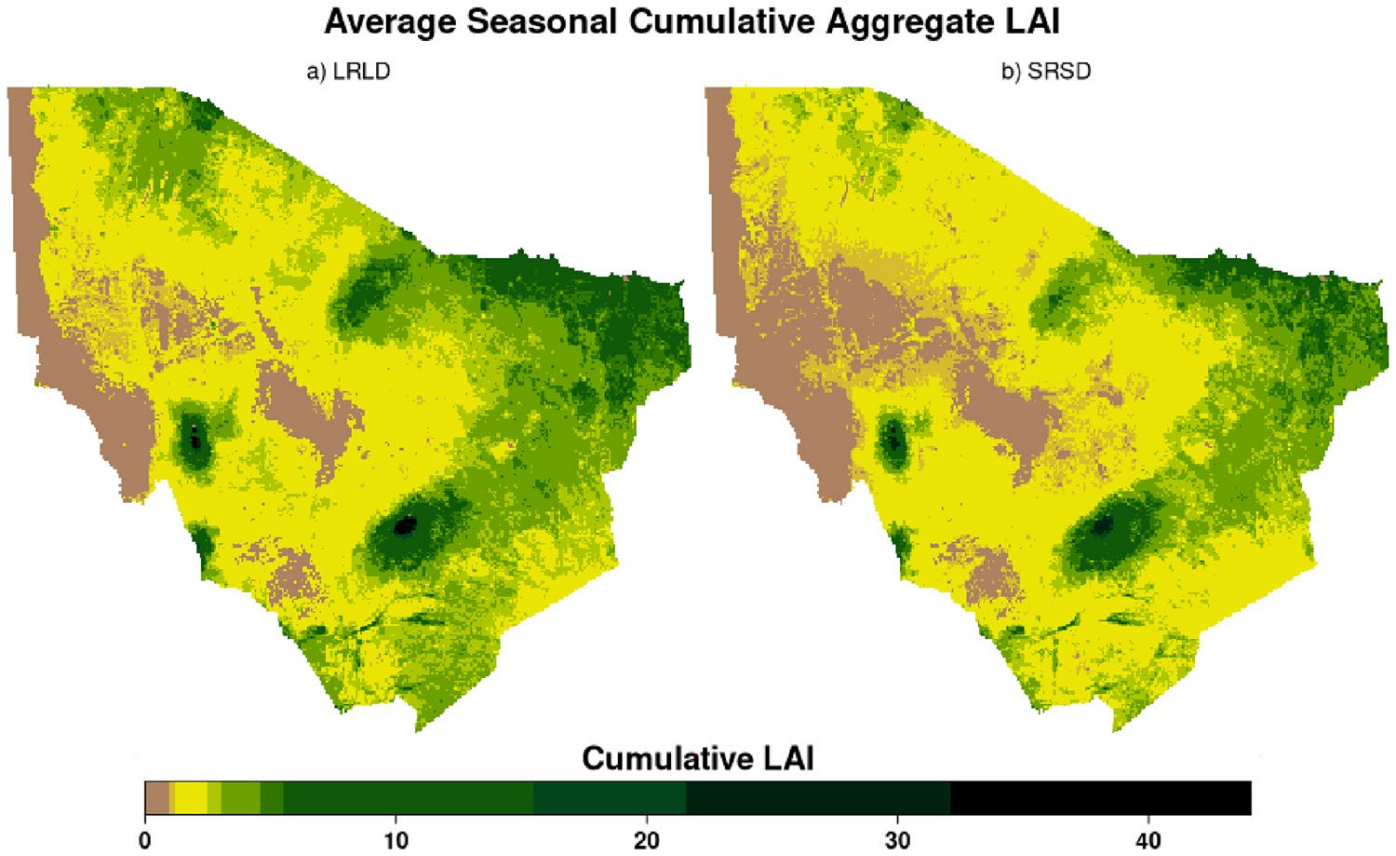
Average seasonal cumulative aggregate leaf area index (LAI_A_) for Marsabit County in Kenya, for the epoch 2003–2021 in (**a**) the long rains long dry season (LRLD; March–September), and (**b**) short rains short dry season (SRSD; October–February). Figure generated in R-Programming (version 4.2.1), using Lattice Package (version 0.10-10).

Using the household buffer described above, we spatially aggregated LAI_A_ to compute the forage index based on LAI_A_. The 8-day household buffer aggregate data was used to compute the monthly average estimates, then cumulated per season for the LRLD and SRSD periods for the years 2003–2021 (Fig. 2). Subsequently, anomalies were derived using z-score standardization. Standardizing vegetation indices is crucial as it ensures the uniformity of data across diverse regions and timeframes, ultimately enhancing the reliability, comparability, and accuracy of the data employed in IBLI and drought modeling^6,47,48^.

##### Partitioned leaf area index

Following the methods proposed in Kahiu and Hanan^44^, the MODIS LAI_A_ was partitioned into woody and herbaceous LAI constituents, denoted as LAI_W_ and LAI_H_ respectively, together denoted as partitioned LAI (LAI_P_; https://sites.google.com/view/partitioned-modis-lai/home)^49^. This update to LAI_P_ uses the preprocessed 8-day MODIS Collection 6.1 (MCD15A2Hv061) at 500 m resolution described above, for the implementation of the partitioning approach which requires three key input parameters: (1) EO based LAI_A_ estimates; (2) woody cover (WC; τ_w_); and (3) potential maximum in-canopy LAI (*LAI*_W*max_). The EO based LAI_A_ was available from MODIS, WC from a woody cover product centered around year 2005^50^, while *LAI*_W*max_ is based on an allometric relationship between in situ in-canopy LAI measurements from Africa and precipitation. Other important details and assumptions are detailed in Kahiu and Hanan^44^.

**Figure 3:**
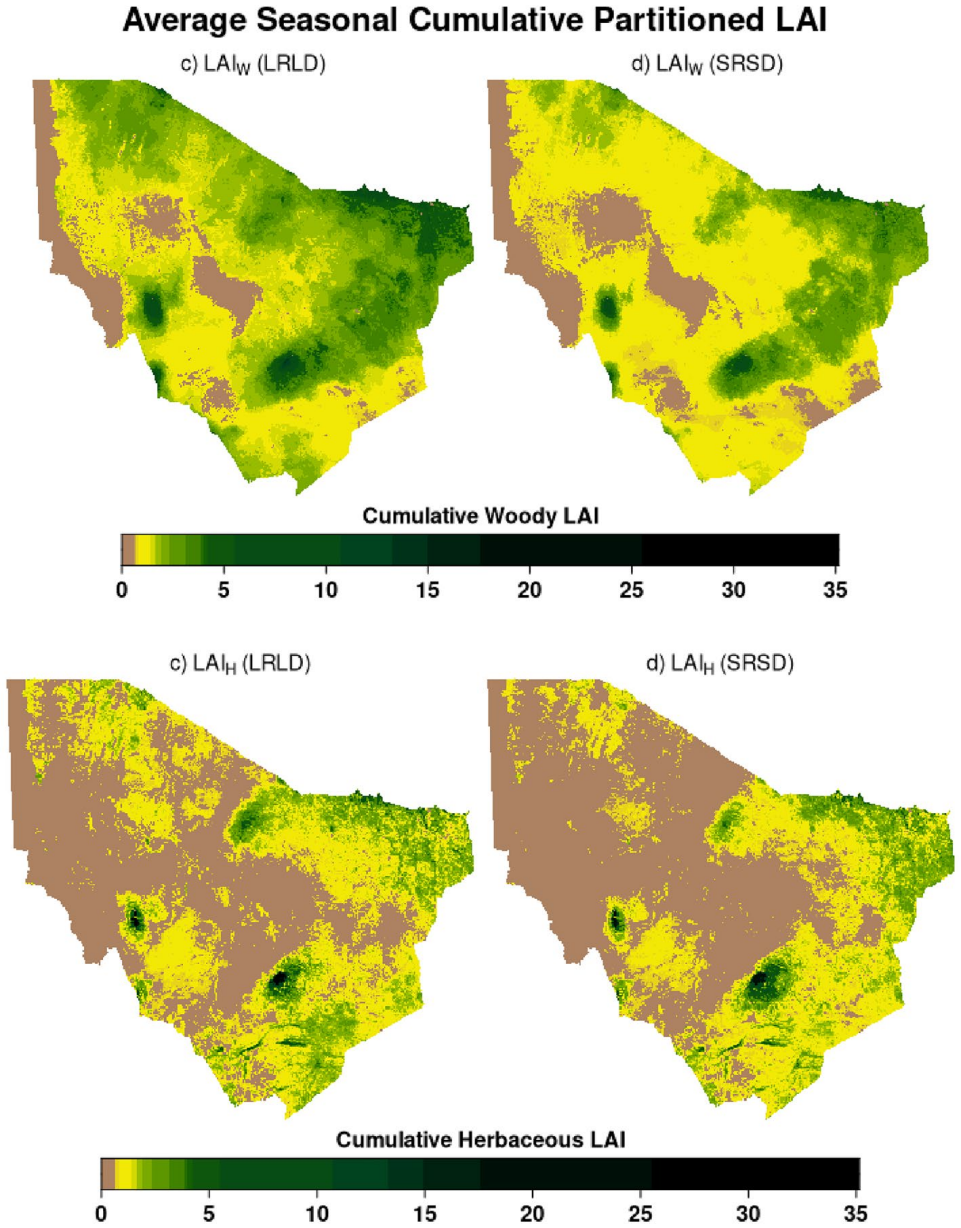
Average seasonal cumulative partitioned leaf area index for Marsabit County in Kenya, for the epoch 2003–2021 in (**a**) woody LAI for the long rains long dry season (LRLD; March–September), and (**b**) woody LAI for the short rains short dry season (SRSD; October–February), (**c**) herbaceous LAI for the long rains long dry season and (**d**) herbaceous LAI for the short rains short dry season. Figure generated in R-Programming (version 4.2.1), using Lattice Package (version 0.10-10).

To derive household level LAI_H_ and LAI_W_, the household buffer approach described above was used to spatially aggregate the 8-day data. The 8-day household buffer aggregate data was used to compute the monthly average estimates then cumulated per season for the LRLD and SRSD periods for the years 2003–2021 (Fig. 3). Subsequently, anomalies were derived using z-score standardization.

##### Normalized difference vegetation index

To align with the 8-day MODIS Collection 6.1 LAI data at 500 m spatial resolution utilized in our study, for which readily available preprocessed data were lacking, we employed the MODIS Version 6.1 Normalized Difference Vegetation Index (NDVI) data from Terra (MOD13A1) and Aqua (MYD13A1) satellites. These datasets were available every 16 days at a 500 m pixel spatial resolution. The 16-day product composites the highest-quality pixel values from all acquisitions within the 16-day period, considering factors like low cloud cover, favorable view angles, and the highest NDVI values. Given the 8-day difference between these two products, we did a temporal compositing to generate an 8-day NDVI product, which matches the MODIS LAI data. The NDVI preprocessing procedure was based on the LAI smoothing approach as outlined in Kahiu and Hanan^44^. This involved employing quality flags to filter out pixels contaminated by clouds, addressing gaps in data for missing dates and pixels, and applying data smoothing techniques to minimize the inherent noise. For data smoothing, we also implemented the robust spline smoothing algorithm implemented in Python^45^.

**Figure 4:**
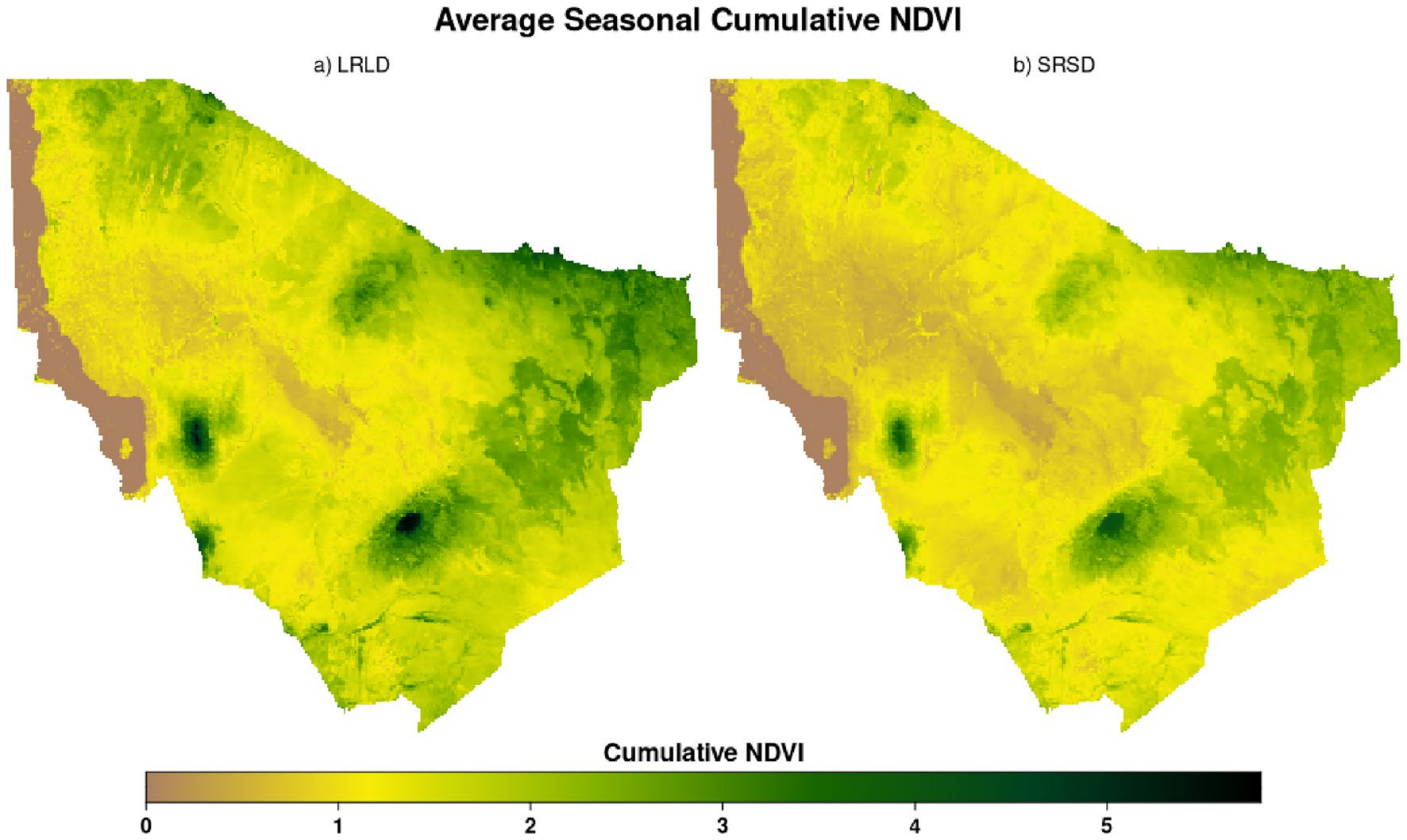
Average seasonal cumulative NDVI for Marsabit County in Kenya, for the epoch 2003–2021 in (**a**) the long rains long dry season (LRLD; March-September), and (**b**) short rains short dry season (SRSD; October–February). Figure generated in R-Programming (version 4.2.1), using Lattice Package (version 0.10-10).

After preprocessing, we used the household buffer zones to spatially aggregate the NDVI data and compute the household forage index from cumulated NDVI for LRLD and SRSD for the 2003–2021 epoch (Fig. 4), following steps described in the LAI section above.

##### Water availability

Water is a critical resource in pastoral ecosystems, influencing the spatial distribution of both livestock and wild herbivores^51,52^. It plays a pivotal role in guiding the seasonal migration patterns of herds, sometimes intersecting with wildlife migration routes^52^. Water points serve as central hubs, directing the seasonal migration of herds. Pastoralists carefully plan their livestock seasonal movement to ensure foraging areas with access to water, which is particularly crucial during dry seasons and droughts^53^. Certain animal types, such as cattle, are heavily dependent on water. Consequently, the availability of adequate water resources is paramount for ensuring their survival and resilience in the face of extreme dryland weather conditions^54^.

**Figure 5:**
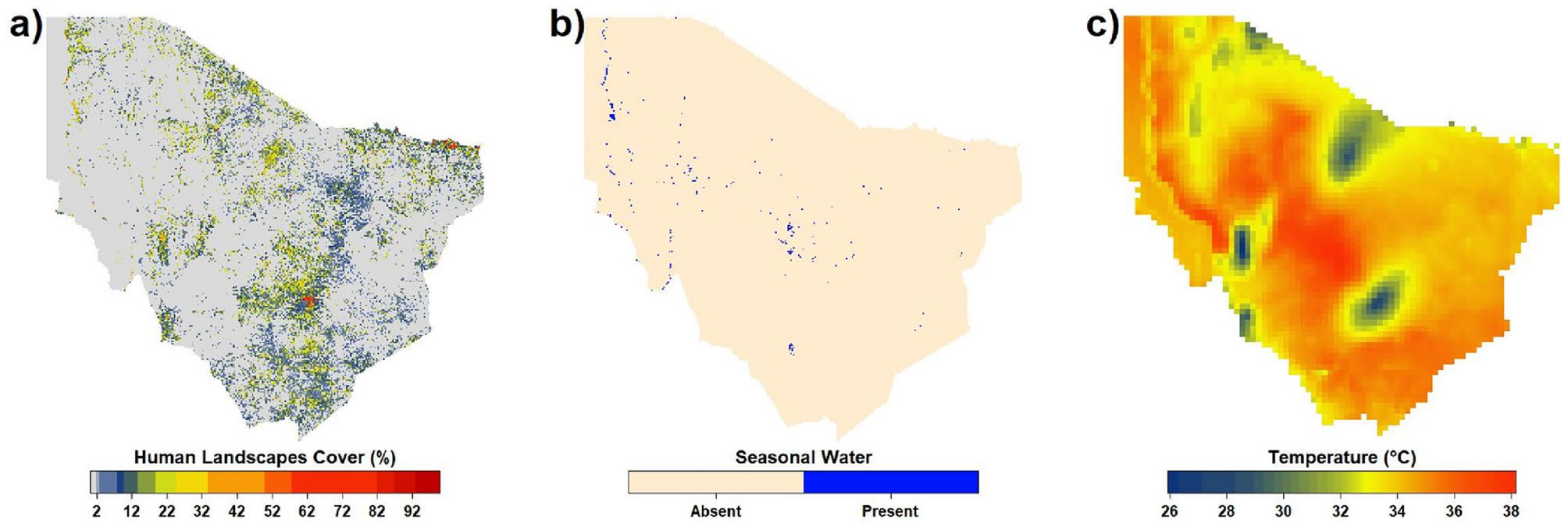
Environmental Variables for Marsabit County used in the analysis including (**a**) Human Landscapes which combines built up and cultivated areas, (**b**) Seasonal surface water resources, and (**c**) Average maximum monthly temperature for the epoch 2003–2021. Figure generated in R-Programming (version 4.2.1), using Lattice Package (version 0.10-10).

We used Version 3 Copernicus Global Land Cover seasonal inland water fractional cover estimates (Fig. 5b), available at 100 m spatial resolution for year 2019^55^. The data ranges between 0% for non-water pixel and 100% for pixels filled with seasonal water at any time during the year. We used these estimates to compute seasonal water density within the 20 km buffer surrounding the household data. It is noteworthy that although additional data on water sources like shallow wells and boreholes would have been beneficial for our analysis, we found that such data were not available in a format or scale compatible with our geospatial layers.

#### Temperature

Climatic variability and weather patterns play a key role in survival and distribution of livestock. Various livestock types have optimal temperature ranges outside which may have adverse impacts on the animal health and performance. Extreme cold and hot temperatures can impact disease susceptibility, threatening livestock health and survival^56^.

Here we used monthly temperature maxima from the TerraClimate global dataset for the epoch 2003–2021 (Fig. 5c). The data comprises monthly temperature maximum estimates for global terrestrial surfaces, available at a spatial resolution of ~ 4 km^57^. This gridded dataset integrates the higher spatial attributes from WorldClimV2 with temporal attributes from CRU Ts4.0, utilizing climatically aided interpolation techniques, to create a high-spatial resolution dataset that covers a broader temporal record^57,58^.

To determine the household level temperature anomalies as an indicator of climate variability, we used the household buffer approach to compute the seasonal average temperature across the LRLD and SRSD periods for the years 2003–2021. Subsequently, anomalies were derived using z-score standardization.

#### Human landscapes

Land use and cover types, crucial determinants of livestock populations and herding practices, can significantly affect pastoralism in Africa. Traditional pastoralists, practicing nomadic and transhumant herding, rely on mobility to access better forage and water resources during droughts. However, land fragmentation, often driven by expanding settlements and agricultural farms, disrupts pastoral migration corridors and movement patterns^59^. This results in smaller, isolated land parcels, reducing available grazing areas and intensifying resource competition among various land users^60,61^. In this analysis, we employed EO derived human landscapes, encompassing built-up and cultivated areas, as a proxy for land fragmentation. These landscapes encroach upon traditional grazing lands, impacting the mobility and livelihoods of pastoral communities and challenging their traditional drought coping mechanisms and survival strategies^60,61^.

To account for the influence from human population and land fragmentation in our models, we used a recently developed EO product from the Copernicus Global Land Service, Collection 3 Land Cover 100 m dataset for the year 2019 (Fig. 5a), available at a spatial resolution of 100 m. These estimates are derived from PROBA-V satellite observations and ancillary datasets^55^. We created the human landscapes layer by summing fractional cover estimates for cultivated and built-up areas. To determine the extent of human landscapes within the 20 km buffer surrounding the household data, we calculated the density by dividing the total human landscapes area divided by the 20 km radius buffer feature.

### Analysis models

#### General forage models

We implemented three competing forage type models: (1) NDVI forage model, (2) aggregate LAI model (LAI_A_), and (3) the partitioned LAI model (LAI_P_) which included separate woody (LAI_W_) and herbaceous (LAI_H_) forage estimates. These forage proxies derived from vegetation indices were integrated with other relevant environmental variables, including water availability, temperature, and the extent of human landscapes, to elucidate the factors influencing livestock mortality rates in Kenyan pastoral rangelands, as summarized in Table 3

**Table 3:** Aggregate and partitioned models used for the herbivory analysis using random forest model.

Response variable (livestock mortality rates in TLUS)	Explanatory variables		
	Aggregate NDVI model (NDVI)	Aggregate LAI model (LAI_A_)	Partitioned LAI model (LAI_P_)
1. Aggregate Livestock	NDVI + Human Landscapes + Water availability + Temperature + Seasonality	LAI_A_ + Human Landscapes + Water availability + Temperature + Seasonality	LAI_H_ + LAI_W_ + Human Landscapes + Water availability + Temperature + Seasonality
2. Cattle			
3. Camels			
4. Shoats (Sheep and Goats)			

LAI_A_-Aggregate leaf area index (LAI); LAI_H_—herbaceous LAI; LAI_P_ – Partitioned LAI; LAI_W_—Woody LAI; NDVI—Normalized Difference Vegetation Index; TLUS-Tropical livestock units.

#### Regression analysis using random forest modelling

To conduct the regression analysis Random Forest Models (RFM) were implemented using the ‘*randomForest’* package in R-Programming^62,63^. RFMs comprise an ensemble of machine learning algorithms that are widely used for both classification and regression analysis^64^. It builds multiple decision trees during training and combines their outputs to improve predictive accuracy and reduce overfitting. In classification tasks, RFM typically uses majority voting among the trees to make predictions, while in regression analysis, it averages the predictions from individual trees. Their robustness is enhanced through feature randomization by considering only a subset of features at each split in the decision tree, to ensure the correlation between trees is reduced and model's generalization is improved.

Randomness plays a crucial role in the RF algorithm, introduced through bootstrapping (randomly sampling subsets of data with replacement) and feature randomization (randomly selecting subsets of features at each split)^64^. To ensure repeatability and optimize our RFMs, we carefully controlled these sources of randomness by setting up 50 random seeds, with results from the 50 models averaged to generate the final results^62,63^.

## Results

### Livestock mortality types

**Figure 6:**
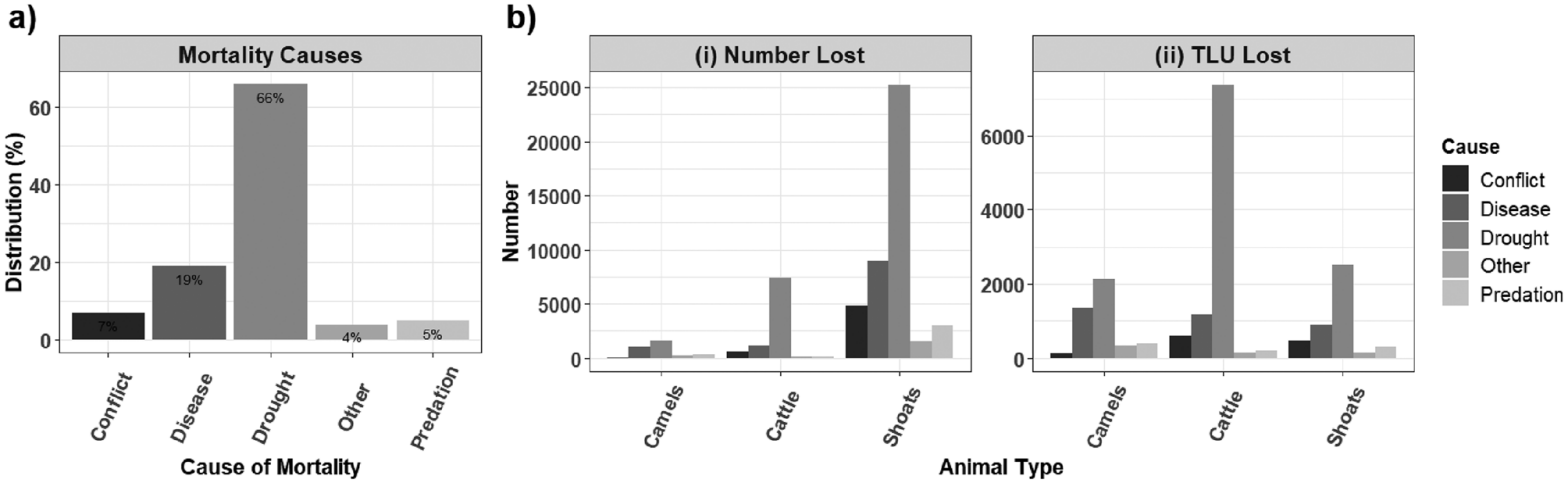
Descriptive analysis of livestock mortality causes, and livestock types affected in Marsabit county, (**a**) causes of livestock losses as a percent of total across all livestock types, (**b**) livestock losses for each type expressed in (i) number of affected, and (ii) Tropical Livestock Units. Figure generated in R-Programming (version 4.2.1), using ggplot2 Package (version 3.5.1).

In African pastoral systems, livestock mortality stems from multiple factors, including drought, wildlife predation, disease, and inter-communal livestock raids. In Marsabit County, northern Kenya, our analyses revealed that drought related mortality accounted for the majority (66%) of livestock losses (Table 1 and Fig. 6a). Prolonged droughts create water and forage shortages, malnutrition, and weakened immunity, rendering livestock susceptible to diseases, consequently increasing mortality^36,39^. Disease outbreaks ranked as the second (19%) leading cause of livestock mortality in the region, particularly during the dry season when livestock congregate around limited foraging and water sources, facilitating disease transmission^32^.

While drought and disease persist as predominant factors contributing to mortality in specific animal species, their impact varied significantly among different types (Fig. 6b). In terms of individual numbers, shoats exhibited the highest vulnerability to mortality from both drought and disease causes (Fig. 6b). However, when considering livestock population in TLUs, cattle emerged as the most significant loss experienced by pastoralists during drought, while camels were the most susceptible to diseases, Fig. 6b (ii).

### Seasonal distribution of livestock mortality across environmental variables

**Figure 7:**
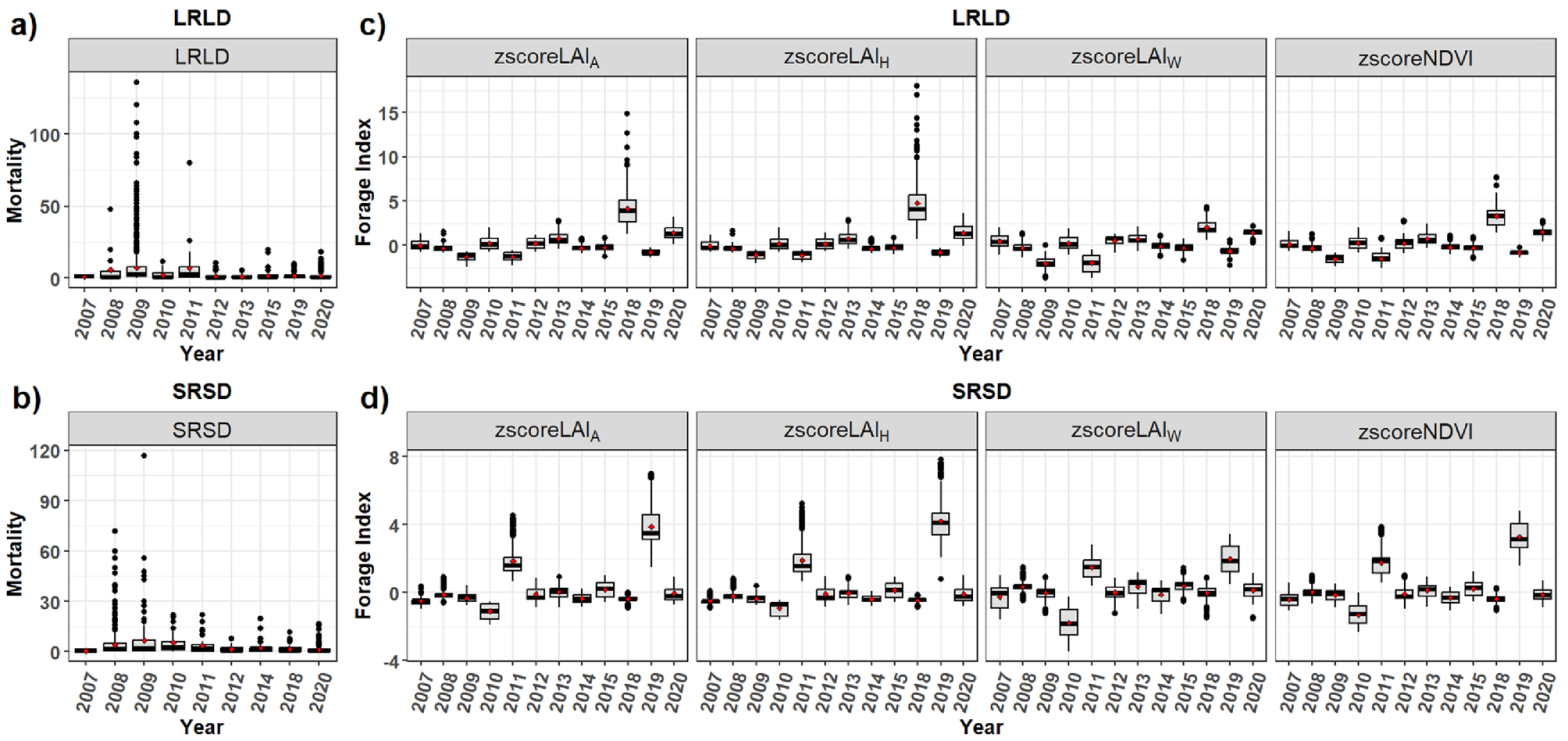
Distribution of livestock mortality during 2008–2021 from drought and disease and the corresponding forage indices across the years in Marsabit County, Kenya. (**a**) Long-term average mortality during the long rains and long dry season, (**b**) Long-term average mortality during the short rains short dry season and the corresponding forage indices (the z-scores) in (**c**) and (**d**) for aggregate LAI (zscoreLAI_A_), Herbaceous LAI (zscoreLAI_H_), woody LAI (zscoreLAI_W_) and Normalized Difference Vegetation Index (zscoreNDVI). Note missing years in the mortality time series in (**a**) and (**b**) correspond to years when mortality data was unavailable. Figure generated in R-Programming (version 4.2.1), using ggplot2 Package (version 3.5.1).

Extreme forage scarcity events aligned with high livestock mortality patterns in Marsabit from 2008 to 2021 (Fig. 7). Notable low forage index values during the long rain season of 2009, were prominently reflected across all the forage indices, which match the high livestock mortality in that period. The distribution patterns of livestock mortality across different ranges of predictor variables are shows in Fig. A1 in Supplementary materials).

#### Overview of performance of all models

To evaluate the effectiveness of our RFMs, we employed model-derived variable importance metrics and calculated the Root Mean Squared Error (RMSE) and R-squared (R^2^) values across all models. RFMs commonly offer insights into variable importance, indicating the extent to which each predictor contributes to minimizing prediction errors. This importance can be expressed either as a percentage or as a ranking among variables. RMSE measures the average magnitude of the model's prediction errors, while R^2^ quantifies the proportion of variance in the dependent variable that is explained by the independent variables in the model.

Overall, the partitioned forage models demonstrated superior performance compared to the aggregate forage models (Table 4 and Fig. 9). The LAI_P_ forage model consistently exhibited lower RMSE and higher R^2^ values across all livestock types when compared to the LAI_A_ and NDVI models. In the aggregate livestock mortality resulting from both drought and disease, the LAI_P_ model stood out with the lowest RMSE of 5.9 TLUs and a high R^2^ value of 0.83. In comparison, the aggregate models comprising of both the LAI_A_ and NDVI models exhibited higher RMSE values (~ 9.3 TLUs) and lower R^2^ values (~ 0.6), which align with our expectations due to their representation of similar aggregate (combined herbaceous and woody) landscape scale forage estimates. The animal specific statistics are summarized in Table 4.

**Table 4:** Random forest results for the aggregate and separate forage models for livestock mortality in Marsabit County, Kenya.

Model	RMSE	R^2^	Variables	Importance	RMSE	R^2^	Variables	Importance
	1) Aggregate mortality from drought and disease				3) Cattle mortality from drought and disease			
Aggregate models								
Aggregate NDVI	9.33	0.59	Aggregate NDVI	19.9%	13.41	0.61	Aggregate NDVI	16.8%
			Human Landscapes	15.1%			Temperature	13.8%
			Temperature	14.9%			Human Landscapes	12.1%
			Season	14.8%			Seasonal Water	12.1%
			Seasonal Water	14.1%			Season	11.2%
Aggregate LAI	9.30	0.60	Aggregate LAI	19.2%	13.38	0.61	Aggregate LAI	16.7%
			Human Landscapes	15.7%			Temperature	13.5%
			Temperature	15.5%			Human Landscapes	11.9%
			Seasonal Water	13.8%			Seasonal Water	11.9%
			Season	13.4%			Season	10.4%
Partitioned models								
Partitioned LAI	5.88	0.83	Human Landscapes	21.7%	9.07	0.81	Herbaceous LAI	18.1%
			Temperature	20.8%			Woody LAI	17.3%
			Seasonal Water	19.9%			Temperature	17.2%
			Herbaceous LAI	19.5%			Human Landscapes	14.6%
			Woody LAI	19.5%			Seasonal Water	12.4%
			Season	7.4%			Season	0.5%

	2) Camels mortality from drought and disease				4) Shoats mortality from drought and disease			
Aggregate models								
Aggregate NDVI	6.08	0.75	Temperature	8.0%	2.37	0.67	Aggregate NDVI	9.5%
			Aggregate NDVI	6.4%			Temperature	7.4%
			Seasonal Water	5.5%			Human Landscapes	6.8%
			Human Landscapes	4.8%			Seasonal Water	5.6%
			Season	-1.2%			Season	4.6%
Aggregate LAI	6.03	0.76	Temperature	7.9%	2.37	0.67	Aggregate LAI	7.3%
			Aggregate LAI	6.8%			Temperature	6.5%
			Seasonal Water	5.3%			Human Landscapes	6.4%
			Human Landscapes	4.9%			Seasonal Water	5.4%
			Season	-1.5%			Season	3.8%
Partitioned models								
Partitioned LAI	4.43	0.84	Temperature	12.4%	1.65	0.82	Human Landscapes	9.8%
			Seasonal Water	10.3%			Temperature	9.3%
			Human Landscapes	7.6%			Woody LAI	9.2%
			Woody LAI	7.3%			Herbaceous LAI	8.5%
			Herbaceous LAI	6.8%			Seasonal Water	6.8%
			Season	-0.5%			Season	4.5%

**Figure 8:**
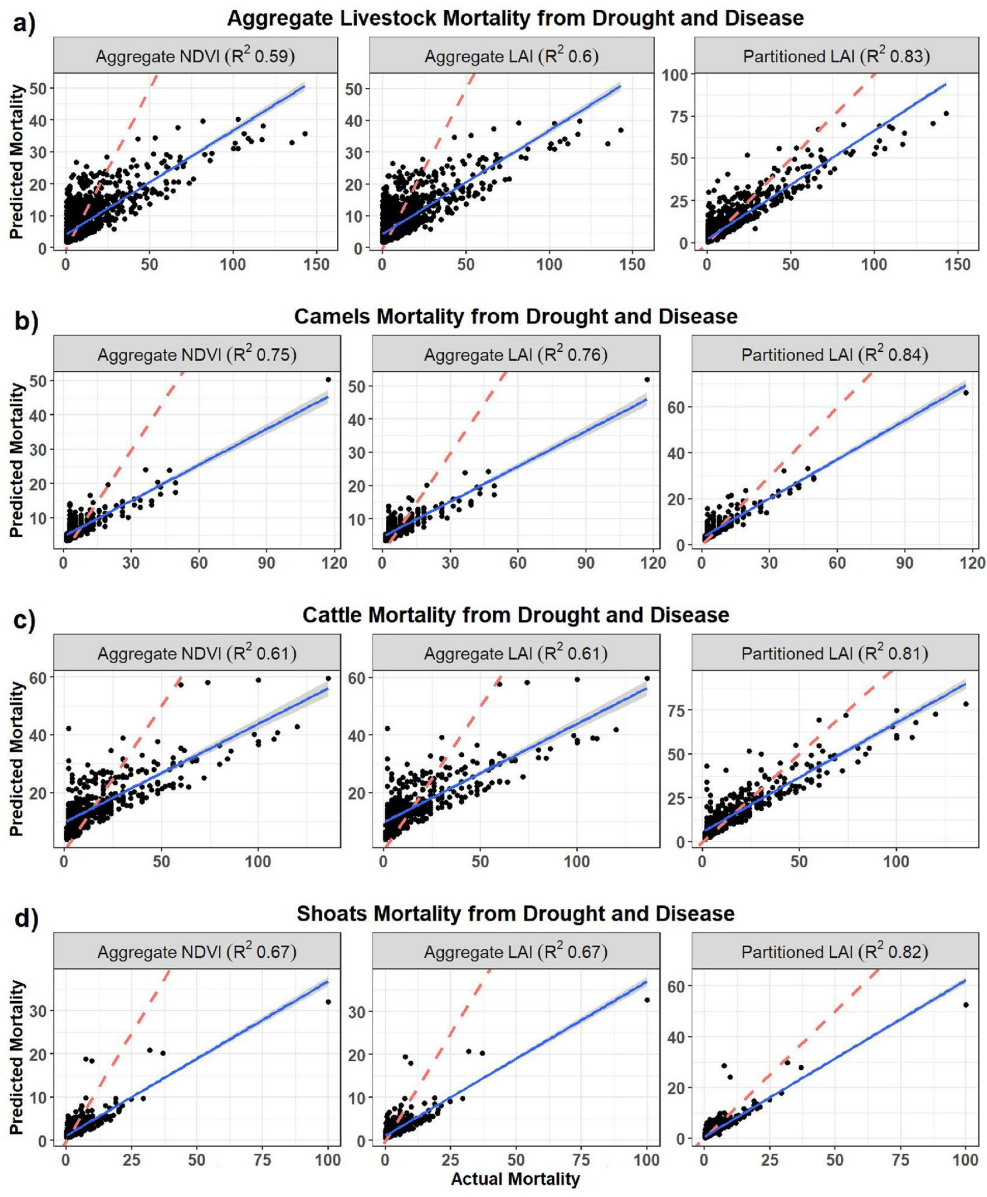
Predicted livestock mortality for aggregate and distinct forage models for (**a**) aggregate livestock mortality (including camels, cattle, goats and sheep), (**b**) shoats (aggregating goats and sheep), (**c**) camel specific and (**d**) cattle specific mortality. Figure generated in R-Programming (version 4.2.1), using ggplot2 Package (version 3.5.1).

The aggregate forage models tended to underestimate the mortality rates across the various animal specific mortality categories and the goodness of fit as shown by R^2^ as shown in Fig. 8a,b (panels 1 and 2). In the all-livestock aggregate mortality (camel, cattle, goats, and sheep) and shoats (b) models, there was generally poor performance in mortality rates lower than 50 TLUs (Fig. 8a,b respectively). Conversely, the partitioned forage model performed better across the various mortality ranges in both the aggregate and animal specific models as evidenced by the higher R^2^ values (Fig. 8, 3rd panel).

The relatively poor performance of the year 2008 short rains followed by a severe drought season during the 2009 LRS, had a significant impact, resulting in exceptionally high mortality rates. These rates appeared as outliers in the data across all the models, but were more pronounced in the animal specific models (Fig. 8b,c).

#### Aggregate livestock mortality models

**Figure 9:**
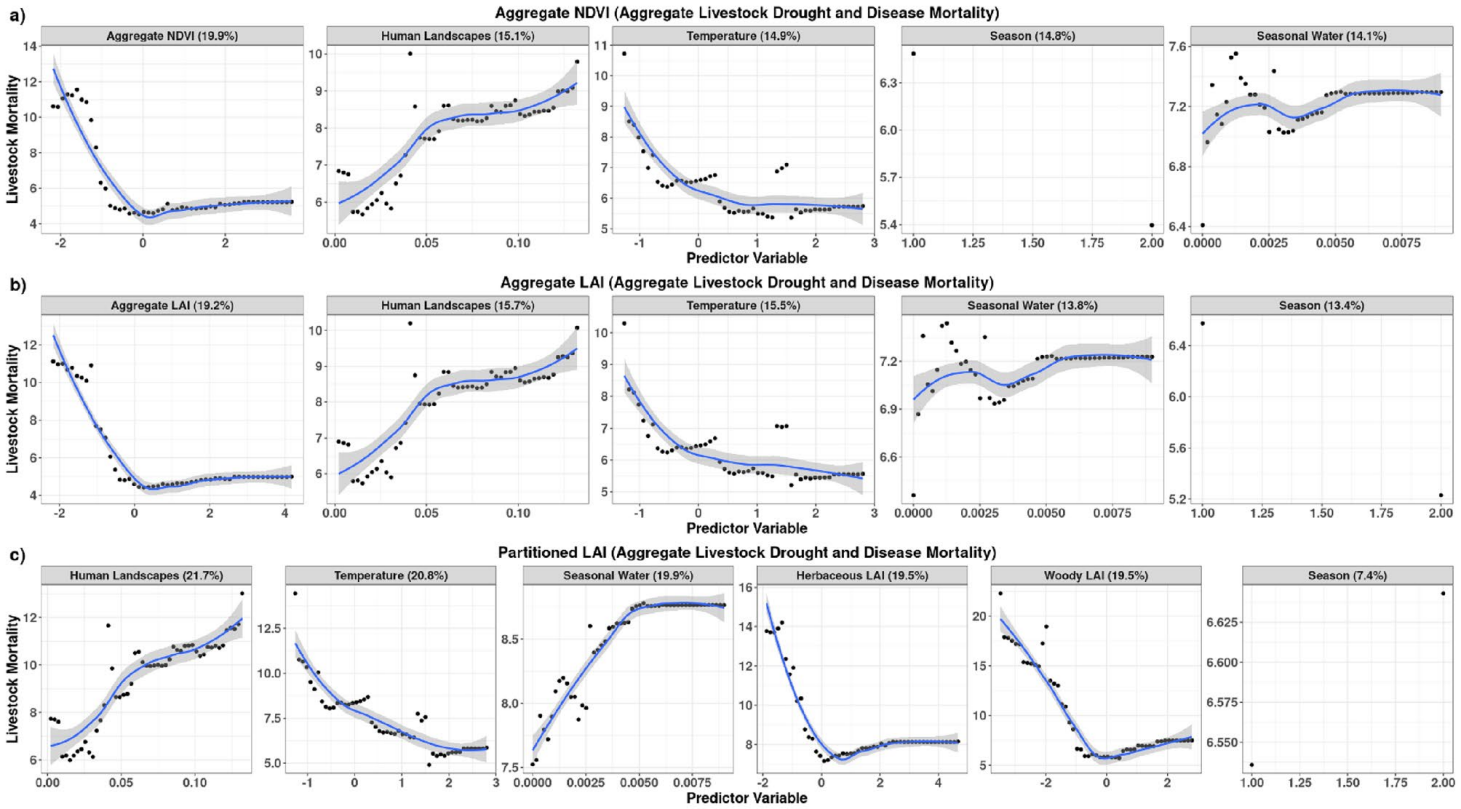
Results showing level of importance and direction of influence for (**a**) aggregate NDVI, (**b**) aggregate LAI and (**c**) partitioned LAI models for explaining aggregate livestock mortality in Marsabit County, Kenya. Percentage values in the header of each variable panel represent variable importance in the models. Figure generated in R-Programming (version 4.2.1), using ggplot2 Package (version 3.5.1).

In the aggregate livestock mortality models, forage availability emerged as the primary determinant of livestock mortality in both the NDVI and LAI_A_ models, contributing 19.9% and 19.2% importance in the respective models (Fig. 9 and Table 4). Conversely, in the LAI_P_ model human landscapes ranked highest with importance at 22% followed by temperature at 21%, water availability at 20%, while both LAI_H_ and LAI_W_ at 19.5%. Grazing (LAI_H_), browsing (LAI_W_) and water resources demonstrated almost equal importance. This observation may underscore the significance of both water and foraging resources in the overall livestock mortality model, or perhaps could suggest a high correlation between both foraging and water resources with rainfall. Water availability and seasonality interchanged positions of importance in the aggregate models, ranging between 13.4 and 14.8% (Fig. 9a,b).

Across all three models, forage availability consistently exhibited a negative correlation with mortality, aligning with the anticipated outcome of increased mortalities when forage availability falls below average levels during drought conditions (Fig. 9a,c). Conversely, the intensity of human activity increased mortality across all models. This could be attributed to increasing density of livestock and associated reduction in accessible grazing lands in locations with higher human populations, potentially leading to increasing competition for grazing resources. However, it’s noteworthy that a positive relationship emerged between mortality and seasonal water availability in all the models. This contradicted our expectation that mortality would decrease in areas with more abundant water resources.

Further, we conducted an analysis for drought only related aggregate mortality for all animals. While there may be subtle changes in the order of importance, the overall influence remained relatively consistent (Fig. A2 and Table A1 in the Supplementary materials), compared to the drought and disease mortality models (Fig. 9).

#### Animal specific mortality models

To understand the impacts of forage availability on browsers and grazers, we ran animal specific models combining drought and disease related mortality. We ran models specific to camels, cattle, and shoats (sheep and goats). Our results showed variations across the models depending on forage model type and level of mortality aggregation or separation (Supplementary materials Sections C–E).

The ranking of significance among the predictor environmental variables, crucial in explaining livestock mortality during drought, varied across models, contingent on the specific forage type and its relevance to distinct animal categories. In general, forage availability consistently emerged as the most influential variable, with species specific models showing the different forage preferences in various animal types. It exhibited a negative correlation with mortality, aligning with the anticipated outcome of increased mortalities when forage availability falls below average levels during drought conditions. Conversely, the intensity of human activity demonstrated a positive relationship with mortality across most models. This could be attributed to the reduction in accessible grazing areas and heightened resource competition in more highly populated and fragmented landscapes. In cattle which are predominantly grazers, LAI_H_ was the most influential factor in the LAI_P_ model, followed by LAI_W_. Conversely, in camels, in the aggregate and partitioned forage models, temperature emerged as the most influential variable, demonstrating a negative correlation with mortality, while the increased importance of LAI_W_ compared to LAI_H_ aligns with the browsing behavior of camels (Table 4). The results in the shoats’ model which combines sheep and goats were more intricate than in the other animal specific models, which may be influenced by the sheep to goat ratio, an important factor that was missing from our dataset. Further details on animal specific model performance are presented in the Supplementary Materials (Sections C–E).

## Discussion

Overall, the partitioned forage models outperformed the aggregate forage models (LAI_A_ and NDVI models; Table 4). The LAI_P_ model consistently exhibited lower RMSE and higher R^2^, indicating a better goodness of fit between the modeled and actual values, across all livestock types when compared to the LAI_A_ and NDVI models. In the aggregate models, both the LAI_A_ and NDVI models had similar performance with comparable RMSE and R^2^ values, indicating no preference as expected. The influence of environmental variables across the models varied, depending on level of mortality aggregation or separation. In cattle which are predominantly grazers, LAI_H_ is the most influential factor in the LAI_P_ model, followed by LAI_W_. Conversely, in camels, the increased importance of LAI_W_ compared to LAI_H_ aligns with the browsing behavior of camels. However, the results in the shoats’ model which combines sheep and goats were more intricate than in the other animal specific models, which may be influenced by the sheep to goat ratio, an important factor missing from our dataset. These findings suggest that by generating distinct estimates for browse and grazing forage through partitioned LAI, has the potential to improve IBLI product design by separating the effect of seasonal fluctuations and long-term variations in woody and herbaceous leaf area, allowing a more precise index of herbaceous and woody forage resources.

Further, our results showed in the majority of the models, the correlation between forage availability indices (NDVI, LAI_A_ and LAI_P_) and livestock mortality rates becomes nearly negligible when the index values are greater than or equal to zero but exhibits a notably strong and negative correlation when the index values are less than zero. This observation suggests that these indices might be most effective in extreme drought scenarios, potentially indicating existence of other influential factors contributing to medium to low drought related livestock mortality, that may need further investigation.

The animal specific LAI_P_ models effectively captured the feeding preferences in various animal types. In cattle which are predominantly grazers^65^, LAI_H_ was the most influential factor in the LAI_P_ model, followed by LAI_W_. Conversely, in camels, the increased importance of LAI_W_ compared to LAI_H_ aligns with the browsing behavior of camels^65^. These findings highlight the significance of distinct role played by browse (LAI_W_) and herbaceous (LAI_H_) foraging resources in ensuring the survival and wellbeing of various livestock species. This distinction is particularly crucial for different livestock species with varying feeding behaviors, as they navigate through changing seasons and years with fluctuating levels of forage production.

We observed a more complex pattern across the mixed sheep and goats (shoats) mortality models. Aggregate forage estimates showed the highest importance across the aggregate (NDVI and LAI_A_) forage models, while human landscape was the most important in the LAI_P_ model. Seasonal water and seasonality were consistently the least important variables across the models (Figs. A6 and Table 4). In the shoats LAI_P_ model we observed a somewhat different pattern between LAI_H_ and LAI_W_ compared to other animal-specific models. Contrary to our expectations in the LAI_P_ model, LAI_W_ ranked higher than LAI_H_. We anticipated that LAI_H_ would have greater significance in the LAI_P_ model since sheep are predominantly grazers, whereas goats though predominantly browsers, mainly feed on shorter shrubs and forbs^66^, which may be captured as LAI_H_ in the partitioned LAI estimates^44^. However, it is noteworthy that the influence of LAI_H_ and LAI_W_ may be contingent upon the ratio of sheep to goats within the mortality data, a crucial factor that was absent from our dataset. This absence might have influenced the observed associations and underscores the complexity of disentangling the impact of these variables on shoat mortality accurately. It is noteworthy that in both LAI_H_ and LAI_W_, mortality initially rose with diminished forage availability, stabilized under typical normal forage conditions, then rose once more with increasing forage availability before reaching a plateau.

Livestock distribution, survival and wellbeing is intrinsically dependent on the climate and its variability^67,68^. Livestock species exhibit optimal temperature ranges, and deviations from these ranges can influence disease prevalence, thereby affecting livestock health and survival^56^. Moreover, temperature fluctuations can disrupt vegetation growth, especially during droughts^69^. In our cattle and camel mortality models, we consistently observed a negative correlation between temperature and mortality. Temperature negatively correlated with cattle and camel mortality, with higher mortality rates occurring in colder conditions, indicating increased susceptibility to diseases. While camels have a broad temperature tolerance range, their optimal conditions typically fall within a warm to hot range^70,71^. Although they can withstand brief cold periods, prolonged cold conditions may compromise their immunity, rendering them more susceptible to diseases, particularly respiratory infections^72^.

While our models generally aligned with expected patterns in explaining livestock mortality associated with drought, several limitations must be acknowledged, which could have introduced uncertainties and unexpected results. During the household surveys for mortality data collection in Marsabit, respondents were asked to recall specific livestock related events, including losses, intakes, offtakes, slaughters, and births, all within the context of the 12 months preceding each survey and with reference to a specific month of the year^34^. This method almost certainly introduced errors and inaccuracies in the reported mortality statistics. Although we made efforts to identify and eliminate apparent data duplications or inconsistencies, some incorrect data may have persisted, potentially affecting our analysis results. Our approach to aggregating mortality, including reported disease and drought causes as a measure of drought related mortality, is logical. We contend that droughts within pastoral systems restrict the availability of forage and water resources, thus results into a decrease in both the quality and quantity of nutrition accessible to livestock, thereby heightening their vulnerability to diseases and raising mortality rates^36,39^. However, other factors may contribute to mortality, introducing errors in the final model results. Furthermore, the mortality data only covers a limited region in northern Kenya. To provide more conclusive results, a more representative dataset should be used. An important consideration in selecting data for fitting the IBLI models should involve exploring additional influential factors beyond mortality that might also constrain livestock production. This broader approach will offer a more holistic understanding of the dynamics influencing livestock welfare and productivity in pastoral systems.

Moreover, in the HoA region, droughts can persist for multiple seasons or even years^73^. Consequently, the survival of herbivores, both wildlife and livestock populations, becomes partly reliant on the forage conditions in the preceding season. An already weakened animal population is unlikely to endure a subsequent severe drought. Thus, considering this potential impact, we initially considered incorporating the forage indices from the previous season in our analysis. However, we found that this inclusion had minimal influence on the results, thus decided to exclude this variable from our models.

Our partitioned LAI estimates were derived based on a woody cover product centered around the year 2005 to constrain the woody LAI^44^. While this approach should generally capture the overall vegetation patterns due to the slow changing nature of woody vegetation, rapid changes in woody cover may not be fully represented, potentially leading to inaccuracies in the partitioned LAI estimates. This could, in turn, affect the overall mortality analysis results. Our LAI partitioning method may also struggle to account for very short shrubs, typically browsed by smaller livestock, thus may introduce complexities in the analysis of smaller ruminants.

Non-physical factors, such as socioeconomic welfare of households, land management practices, social networks, government policies and support, and livestock breeds, can influence livestock vulnerability and resilience to drought^74^. However, these data are challenging to obtain and were not integrated into the current analysis. This may increase basis risk in IBLI models, although it is still uncertain on whether they are appropriate for inclusion in index insurance models. Additionally, while seasonal water availability serves as a suitable indicator of available water resources, other sources such as shallow wells, boreholes and tapped water may also play significant roles in determining drought related livestock mortality. These additional parameters may introduce uncertainties into our models.

### Supplementary Information

Supplementary Information.
