# Supplementary material for: Leveraging browse and grazing forage estimates to optimize index-based livestock insurance
Source: Sci Rep. 2024 Jun 27;14:14834. doi: 10.1038/s41598-024-62893-4 (PMC11211467; doi:10.1038/s41598-024-62893-4)
Supplement: Supplementary file 1 — Supplementary Information. [file 41598_2024_62893_MOESM1_ESM.docx]

Leveraging browse and grazing forage estimates to optimize index-based livestock insurance.

Kahiu, Njoki *^1, 2^; Anchang J.^1^; Alulu, V.^2^; Fava F.P.^2, 3^; and Jensen N.^2,4^ Hanan N.P.^1^

^1^New Mexico State University, USA; ^2^International Livestock Research Institute (ILRI); ^3^Università degli Studi di Milano, Department of Environmental Science and Policy (ESP); and ^4^University of Edinburgh, Scotland

*nkahiu@nmsu.edu

Supplementary Materials

1. Distribution patterns of livestock mortality across different predictor variables.

To understand the distribution patterns of livestock mortality across different ranges of predictor variables, we employed the *bplot*.xy function from the *Fields* package in R-programming. We binned mortality (from drought and disease) on the environmental variables used in the analysis. These boxplot summaries offer a rough insight into how mortality is conditionally distributed across the various predictor variable ranges (Figure A 1)

Forage availability indicators (LAI_A_, LAI_H_, LAI_W_ and NDVI) and human landscapes demonstrate a negative relationship with mortality (Figure A 1a-d, f.) Temperature initially displays high mortality in the negative anomaly range, normalizing in the 0-1 anomaly range, and subsequently increasing in the higher positive anomalies (Figure A 1 e). Additionally, mortality decreases as seasonal water density rises, indicating a negative relationship with available surface water (Figure A 1 g).


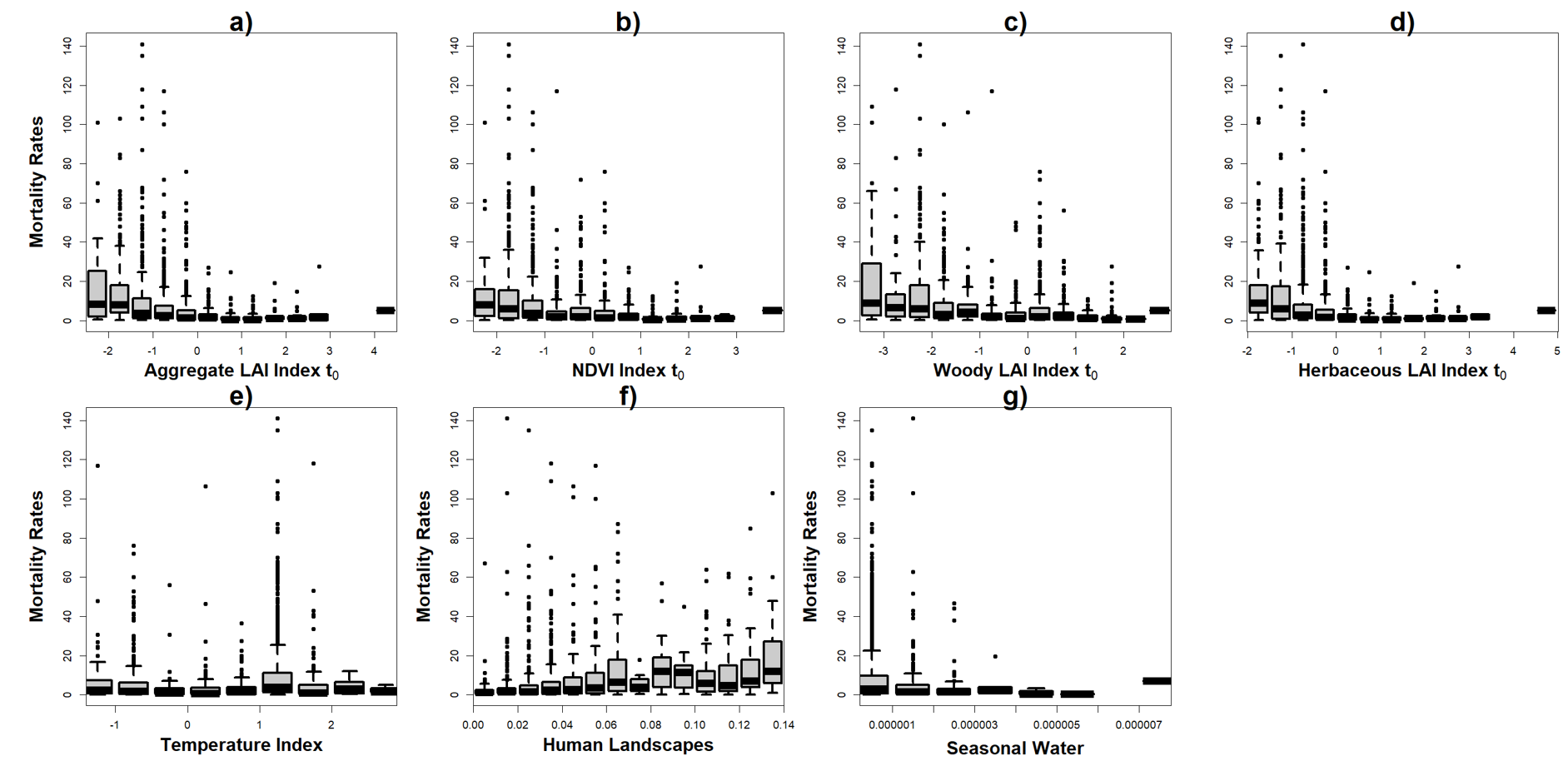


Figure A 1: Distribution of livestock mortality in tropical livestock units across the various predictor variables used in the analysis. a) Aggregate LAI, b) Aggregate NDVI, c) Woody LAI, d) Herbaceous LAI, e) Temperature, f) Human landscapes which includes built up and cultivated areas and g) Seasonal water. Note: a-e variables are normalized through z-score standardization, while f-g represent density, i.e., TLUs/km^2^. Figure generated in R-Programming (version 4.2.1), using ggplot2 Package (version 3.5.1)

1. Results for the aggregate livestock mortality from drought only.

Here we show the results from drought only related aggregate mortality for all animals. While there may be subtle changes in the order of importance (Figure A 2 and Table A1), the overall influence remains relatively consistent with the drought and disease aggregate mortality, as presented in the main document results (Figure 9).


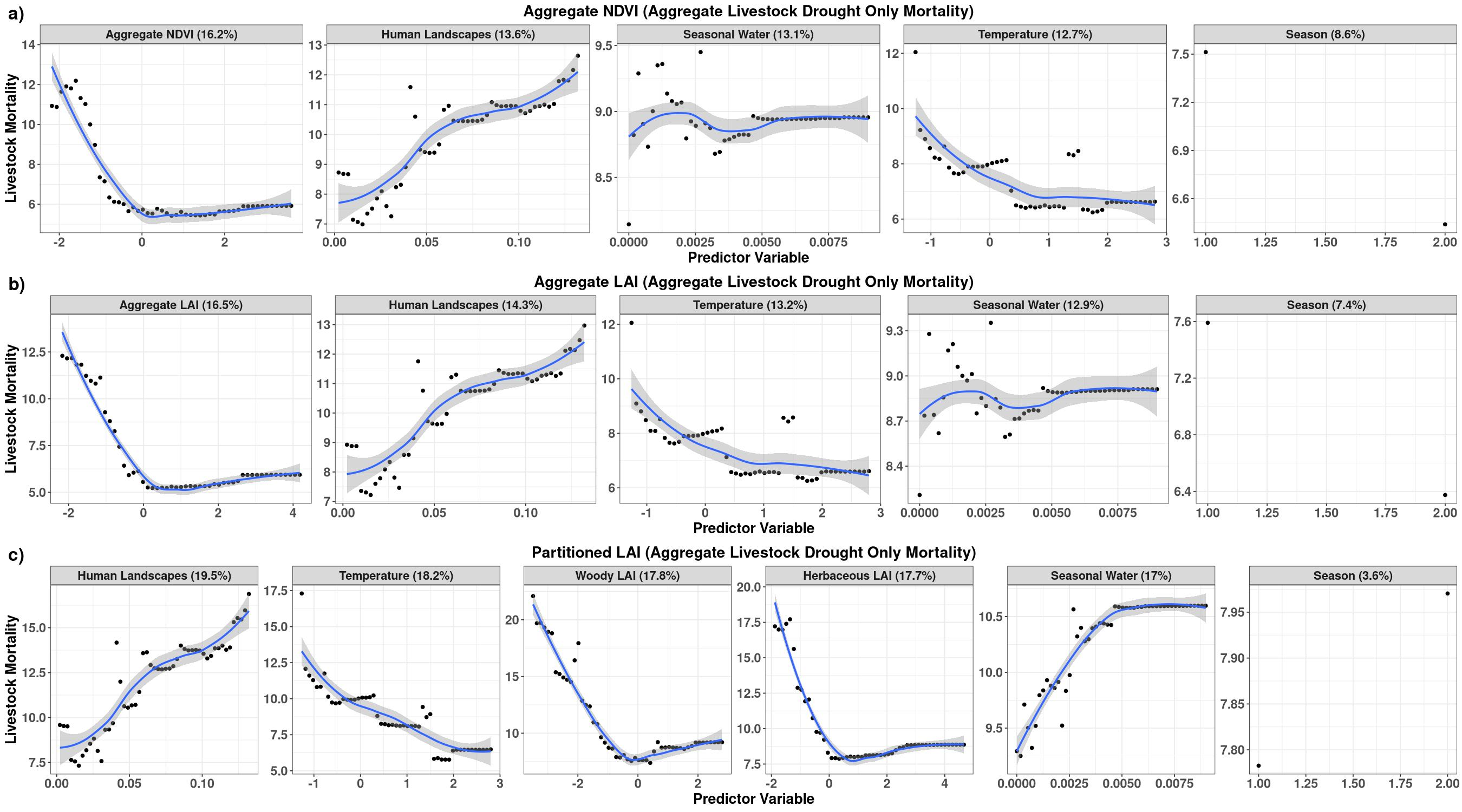


Figure A 2: Results for the aggregate livestock for drought only related mortality from the random forest regression models, for a) aggregate NDVI, b) Aggregate leaf area index and c) partitioned leaf area index. Percentage values in the header of each variable panel represent variable importance in the models. Figure generated in R-Programming (version 4.2.1), using ggplot2 Package (version 3.5.1)

Table A 1: Random Forest results for the aggregate and partitioned LAI models for livestock mortality related to drought only in Marsabit County, Kenya.

1. Camel mortality models

The analysis models combine drought and disease related mortality specific to camels, Figure A 3 and Table 4. In the aggregate and partitioned forage models, temperature emerges as the most influential variable, demonstrating a negative correlation with mortality, exerting an overall impact of ~8% in the aggregate models, and 12.4% in LAI_P_ models (Figure A 3 a-b). Following closely, forage availability contributes significantly in the aggregate forage models, accounting for 6.4% in the NDVI model and 6.8% in the LAI_A_ model. Water availability, human landscapes and seasonality follow in that sequence in both the NDVI and LAI_A_ models. In the aggregate models for both NDVI and LAI_A_, the seasonal water variable exhibits a more gradual slope, indicating a camel's adaptability to areas with both limited and abundant water resources.

In the LAI_P_ model (Figure A 3 c), seasonal water availability assumes the second highest importance, accounting for 10.3%, with human landscapes following at 7.6%. Here, LAI_W_ surpasses the importance of LAI_H_, with contributions of 7.3% and 6.8%, respectively. This observation aligns with the browsing behavior of camels, where woody browse forage estimates (LAI_W_) hold greater significance than herbaceous foraging resources (LAI_H_).


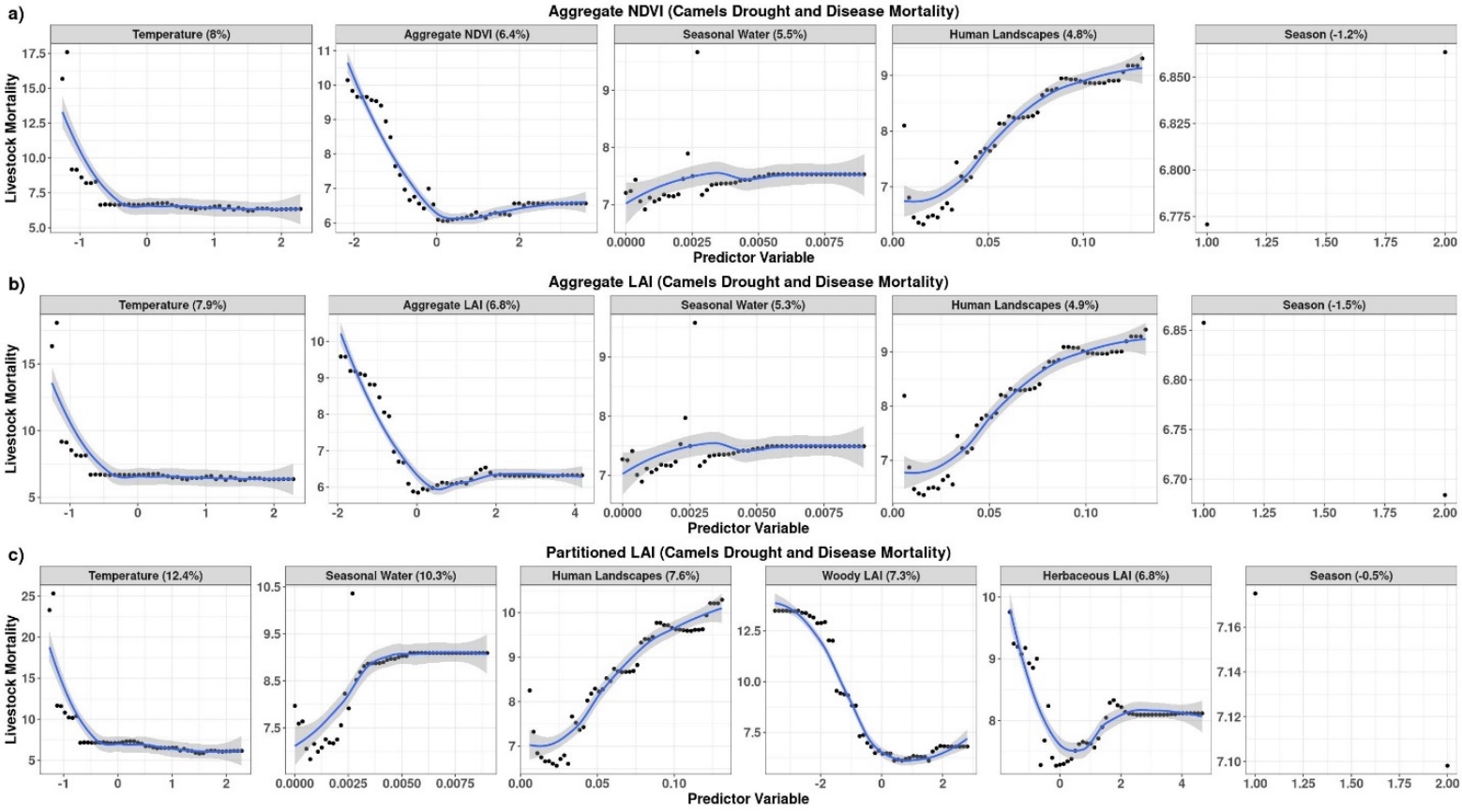


Figure A 3: Results for camels, showing level of importance and direction of influence for a) aggregate NDVI, b) aggregate LAI and c) partitioned LAI models for explaining drought and disease related mortality in Camels in Marsabit County, Kenya. Percentage values in the header of each variable panel represent variable importance in the models. Figure generated in R-Programming (version 4.2.1), using ggplot2 Package (version 3.5.1)

Our results indicate seasonality ranks lowest in importance across all the camel models, registering a negative influence. Typically, in RF analysis, it's recommended to exclude variables with negative influence to enhance model’s predictive performance ^63^. However, when we excluded the seasonality variable, we noticed an unexpected outcome. The model's performance deteriorated as evidenced by a decrease in importance of other variables (Figure A 4). We hypothesize that the negative importance of the seasonality variable may be mitigated by the presence of other correlated features, such as forage availability, which is inherently dependent on seasonal variations. This interplay among variables might explain why removing seasonality had adverse effects on our model's predictive capabilities.


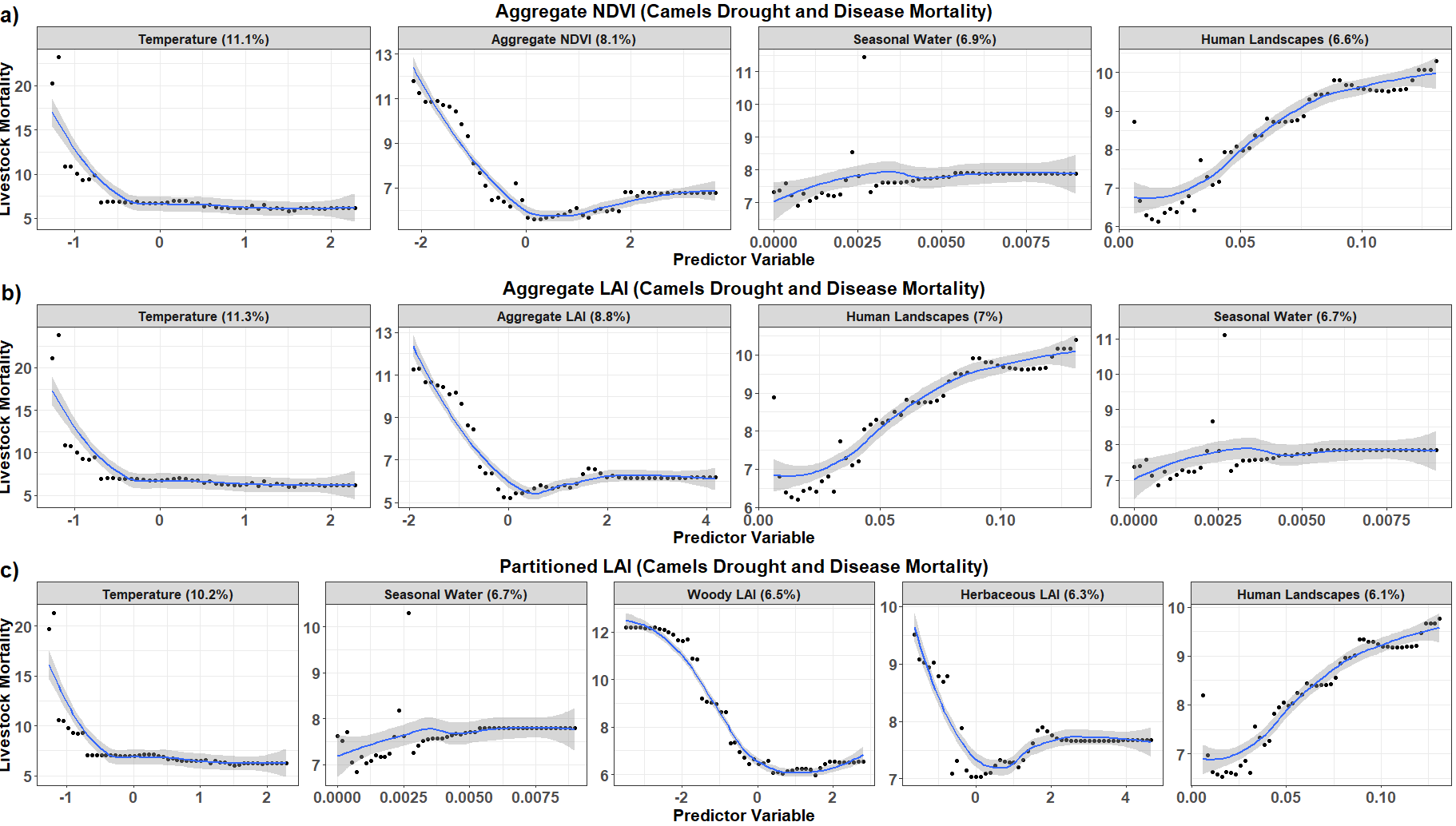


Figure A 4: Results for the camel mortality models after excluding seasonality in the random forest regression models, for a) aggregate leaf area index, b) Aggregate NDVI and c) partitioned leaf area index. Percentage values in the header of each variable panel represent variable importance in the models. Figure generated in R-Programming (version 4.2.1), using ggplot2 Package (version 3.5.1)

1. Cattle mortality models

In the cattle mortality linked to drought and disease, forage availability and temperature emerge as the predominant factors in the three examined models, consistently demonstrating a negative correlation with mortality rates (Figure A 5 a-c and Table 4). In the aggregate models (Figure A 5 a-b), forage availability plays a substantial role, accounting for ~17% of the variation in mortality for both the NDVI and LAI_A_ models. Human landscapes, water availability and seasonality follow in that sequence in both the NDVI and LAI_A_ models.

Considering cattle are predominantly grazers, LAI_H_ is the most influential factor, contributing to 18.1% of cattle mortality in the LAI_P_ model (Figure A 5 c). LAI_W_ ranks second at 17.3%, while temperature ranks third, exerting an influence of 17.2%. Mortality rates are high in negative temperature anomalies and decline in above average temperature ranges, possibly indicating that below normal temperatures could be causing cattle to be more susceptible to diseases, leading to increased mortality.


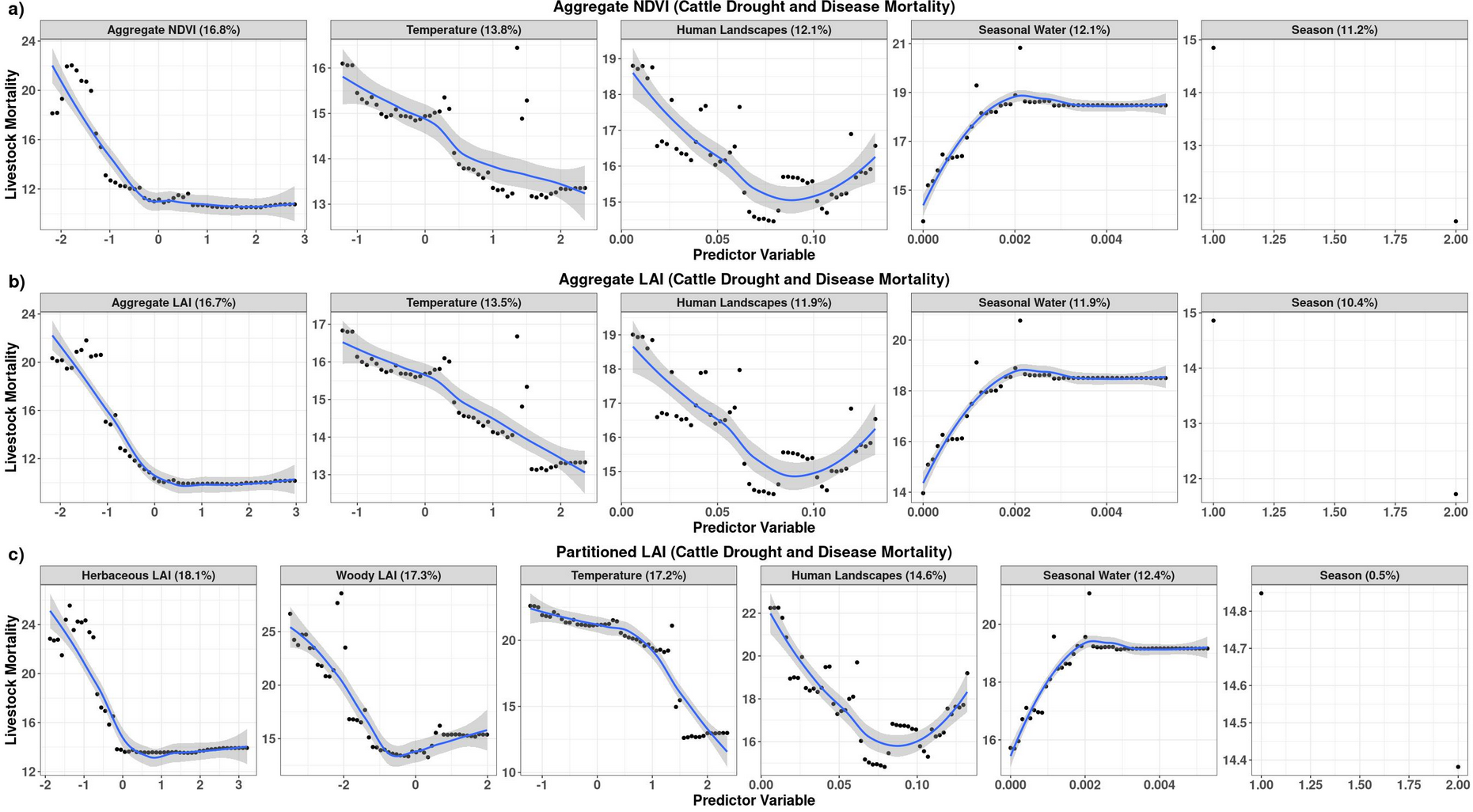


Figure A 5: Results showing level of importance and direction of influence for explaining drought and disease related mortality in Cattle in Marsabit County, Kenya. a) Aggregate NDVI, b) Aggregate LAI and c) partitioned LAI models. Percentage values in the header of each variable panel represent variable importance in the models. Figure generated in R-Programming (version 4.2.1), using ggplot2 Package (version 3.5.1)

Seasonal water yielded unexpected results across the three forage models in relation to cattle mortality. Contrary to our initial expectations, we observed an increase in mortality in areas with higher seasonal inland water sources. This unexpected outcome suggests the presence of other mortality factors following a drought episode. One plausible explanation is the occurrence of flash floods immediately after a drought, which could elevate mortality rates when animals are at their most vulnerable and weakened state, as we have observed in northern Kenya and southern Ethiopia pastoral areas.

Human landscapes rank as the third most important factor after forage and in both aggregate and partitioned models, contributing to ~12% in both NDVI and LAI_A_, and ~15% in the LAI_P_ models. This variable has a parabolic relationship with cattle mortality, Figure 11, a pattern suggesting cattle mortality tends to be high in sparsely populated areas, often characterized by drier ecosystems. Subsequently, there appears to be a normalization of mortality rates in areas with moderate human landscapes. However, mortality rates rise again in regions with higher human landscapes characterized by higher population densities and increased land fragmentation. This increase in mortality may be attributed to a decline in accessible foraging lands and heightened resource competition, which can lead to an increase in livestock losses. This observation underscores the intricate interplay between human settlement patterns, land fragmentation, and livestock mortality in pastoral ecosystems.

1. Shoats (sheep and goats) mortality models

Using various environmental variables to explain mortality from disease and drought in shoats, variable importance is similar in the aggregate forage models, but becomes more intricate in the LAI_P_ model (Table 4 and Figure A 6). Forage availability shows the highest importance in the aggregate forage models accounting for 9.5% in NDVI, 7.3% in LAI_A_ (Figure A 6 a-c and Table 4), while it ranks third and fourth in LAI_P_ model accounting for 9.2% and 8.5% in LAI_W_ and LAI_H_ respectively. Water availability and seasonality consistently demonstrate the least importance across all models.

In the LAI_P_ model human landscapes and temperature as the most important variables in explaining mortality in shoats. Contrary to our expectations in the LAI_P_ model, LAI_W_ ranks higher than LAI_H_. We anticipated that LAI_H_ would have greater significance in the models since sheep are predominantly grazers, whereas goats, while mainly browsers, tend to forage on shorter shrubs and forbs, which may be captured as LAI_H_ in the partitioned LAI estimates ^43^. Nonetheless, it is noteworthy that the influence of LAI_H_ and LAI_W_ may be contingent upon the ratio of sheep to goats within the mortality data, a crucial factor that was absent from our dataset.

In both the NDVI and LAI_A_ models, forage availability exhibits the anticipated negative relationship, where negative anomalies in the forage index indicate below average forage availability, potentially leading to increased mortality. Conversely, in both LAI_H_ and LAI_W_, mortality initially rises with diminished forage availability, stabilizes under normal forage conditions, then rises with increasing forage availability before reaching a plateau. Human landscapes consistently demonstrate a positive relationship with mortality across all the models, indicating the expected reduction in available foraging areas and potential competition with other anthropogenic activities in the human dominated ecosystems. In the aggregate forage models (Figure A 6 a-b), temperature exhibits a more gradual slope, whereas in the LAI_P_ model, a parabolic relationship is evident (Figure A 6 c). The combined sheep and goat mortality may complicate these results due to their different adaptability to environmental conditions and foraging behavior, which may be masked by aggregation of sheep and goats. Perhaps, more nuanced results would emerge in the animal specific models.


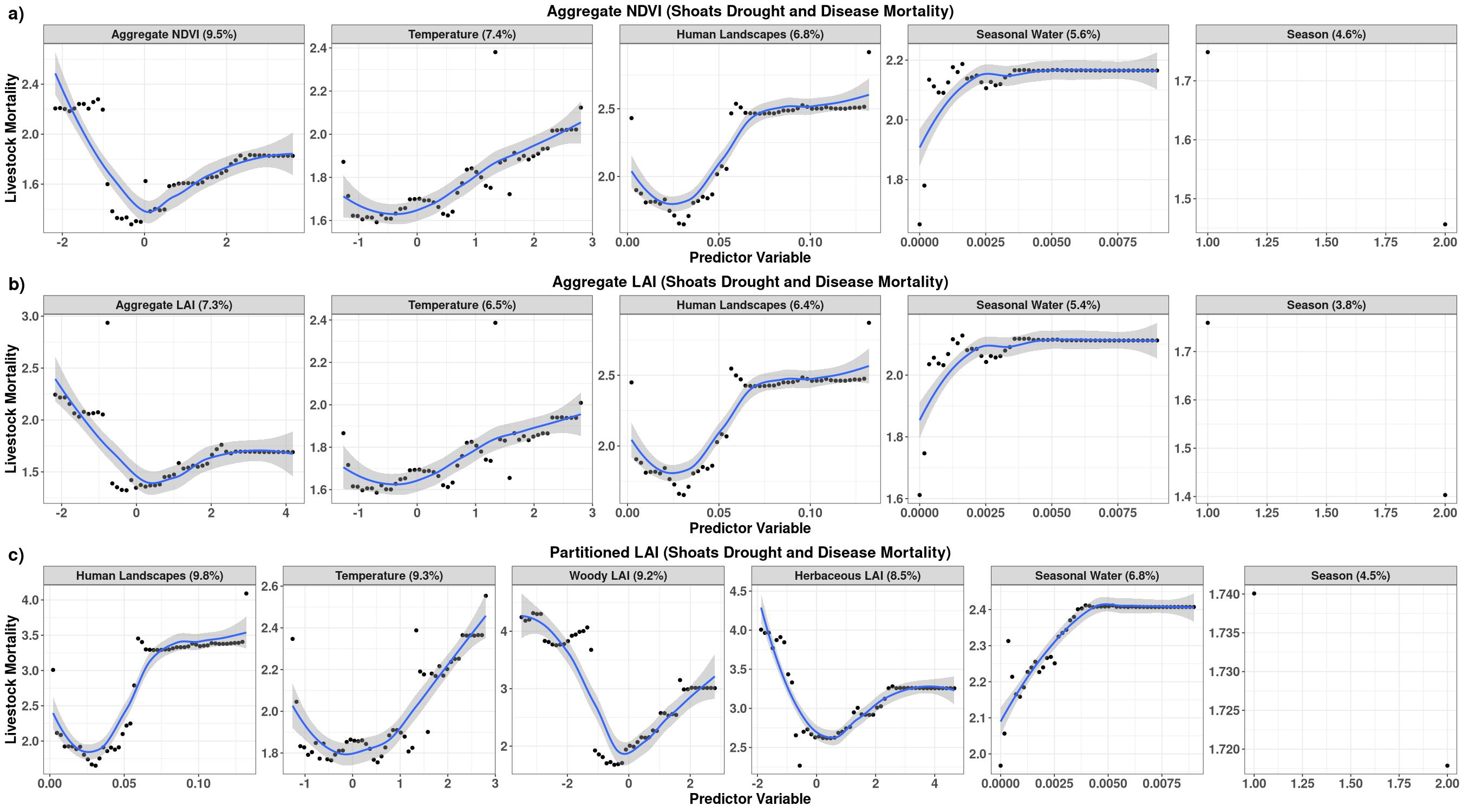


Figure A 6: Results showing level of importance and direction of influence for a) aggregate NDVI, b) aggregate LAI and c) partitioned LAI models for explaining drought and disease related mortality in Shoats (sheep and goats) in Marsabit County, Kenya. Percentage values in the header of each variable panel represent variable importance in the models. Figure generated in R-Programming (version 4.2.1), using ggplot2 Package (version 3.5.1)
